# Safety, efficacy and determinants of response of allogeneic CD19-specific CAR-NK cells in CD19^+^ B cell tumors: a phase 1/2 trial

**DOI:** 10.1038/s41591-023-02785-8

**Published:** 2024-01-18

**Authors:** David Marin, Ye Li, Rafet Basar, Hind Rafei, May Daher, Jinzhuang Dou, Vakul Mohanty, Merve Dede, Yago Nieto, Nadima Uprety, Sunil Acharya, Enli Liu, Jeffrey Wilson, Pinaki Banerjee, Homer A. Macapinlac, Christina Ganesh, Peter F. Thall, Roland Bassett, Mariam Ammari, Sheetal Rao, Kai Cao, Mayra Shanley, Mecit Kaplan, Chitra Hosing, Partow Kebriaei, Loretta J. Nastoupil, Christopher R. Flowers, Sadie Mae Moseley, Paul Lin, Sonny Ang, Uday R. Popat, Muzaffar H. Qazilbash, Richard E. Champlin, Ken Chen, Elizabeth J. Shpall, Katayoun Rezvani

**Affiliations:** 1 Department of Stem Cell Transplantation and Cellular Therapy, The University of Texas MD Anderson Cancer Center, Houston, TX USA; 2 Department of Bioinformatics and Computational Biology, The University of Texas MD Anderson Cancer Center, Houston, TX USA; 3 Department of Nuclear Medicine, The University of Texas MD Anderson Cancer Center, Houston, TX USA; 4 Department of Biostatistics, The University of Texas MD Anderson Cancer Center, Houston, TX USA; 5 Department of Laboratory Medicine, Division of Pathology and Laboratory Medicine, The University of Texas MD Anderson Cancer Center, Houston, TX USA; 6 Department of Lymphoma and Myeloma, The University of Texas MD Anderson Cancer Center, Houston, TX USA

**Keywords:** NK cells, Cancer immunotherapy, Leukaemia, Lymphoma

## Abstract

There is a pressing need for allogeneic chimeric antigen receptor (CAR)-immune cell therapies that are safe, effective and affordable. We conducted a phase 1/2 trial of cord blood-derived natural killer (NK) cells expressing anti-CD19 chimeric antigen receptor and interleukin-15 (CAR19/IL-15) in 37 patients with CD19^+^ B cell malignancies. The primary objectives were safety and efficacy, defined as day 30 overall response (OR). Secondary objectives included day 100 response, progression-free survival, overall survival and CAR19/IL-15 NK cell persistence. No notable toxicities such as cytokine release syndrome, neurotoxicity or graft-versus-host disease were observed. The day 30 and day 100 OR rates were 48.6% for both. The 1-year overall survival and progression-free survival were 68% and 32%, respectively. Patients who achieved OR had higher levels and longer persistence of CAR-NK cells. Receiving CAR-NK cells from a cord blood unit (CBU) with nucleated red blood cells ≤ 8 × 10^7^ and a collection-to-cryopreservation time ≤ 24 h was the most significant predictor for superior outcome. NK cells from these optimal CBUs were highly functional and enriched in effector-related genes. In contrast, NK cells from suboptimal CBUs had upregulation of inflammation, hypoxia and cellular stress programs. Finally, using multiple mouse models, we confirmed the superior antitumor activity of CAR/IL-15 NK cells from optimal CBUs in vivo. These findings uncover new features of CAR-NK cell biology and underscore the importance of donor selection for allogeneic cell therapies. ClinicalTrials.gov identifier: NCT03056339.

## Abstract (web summary)

In the final report of a phase 1/2 trial evaluating allogeneic CD19-specific CAR-NK cells armored with IL-15 in patients with CD19^+^ hematologic malignancies, the therapy was shown to be safe and efficacious with distinct cord blood features associated with response.

## Main

Autologous anti-CD19 CAR-T cells induce remissions in most patients with B cell malignancies^1–4^. However, CAR-T cells have limitations including the cost of therapy, the length of manufacturing and toxicities such as cytokine release syndrome (CRS) and neurotoxicity, which require CAR-T cells to be administered in specialized centers, further limiting access to these potentially life-saving therapies^5,6^. Therefore, there is increasing interest in the development of off-the-shelf cell therapies that are cost effective, safe and potent.

NK cells target cancer cells that downregulate human leukocyte antigen (HLA) class I or express stress markers, and play a critical role in cancer immune vigilance^7–9^. NK cells can be engineered to express a CAR and may be administered without HLA matching with the recipient, thus eliminating the need to produce the CAR product on a patient-by-patient basis^10,11^. We have developed a method to retrovirally transduce allogeneic cord blood unit (CBU)-derived NK cells to express anti-CD19 CAR, IL-15 to enhance their in vivo expansion and persistence, and inducible caspase-9 (iC9) to trigger apoptosis of the CAR-NK cells in the event of unacceptable toxicity (referred to as CAR19/IL-15)^12^. Subsequently, we initiated a study to investigate the safety and efficacy of this strategy in patients with CD19-expressing malignancies, and reported on the dose-escalation portion of the trial^13^. In our study, CAR-NK cell products were manufactured from a different CBU donor for each patient. However, we have shown that from a single CBU we can manufacture hundreds of doses of CAR-NK cells^12^. As inter-donor variability in immune effector function may dramatically impact the likelihood of response^14–18^, it is of paramount importance to define criteria for the selection of optimal CBUs for CAR-NK cell production.

Here, we report on the results of the completed trial and identify CBU characteristics that can be used to select the units most likely to induce a clinical response. Furthermore, we investigated the underlying biological mechanisms for the observed heterogeneity in NK cell potency. Finally, we validated our CBU selection criteria for CAR-NK cell production using multiple preclinical tumor models and CAR targets.

## Results

### Trial design

**Fig. 1:**
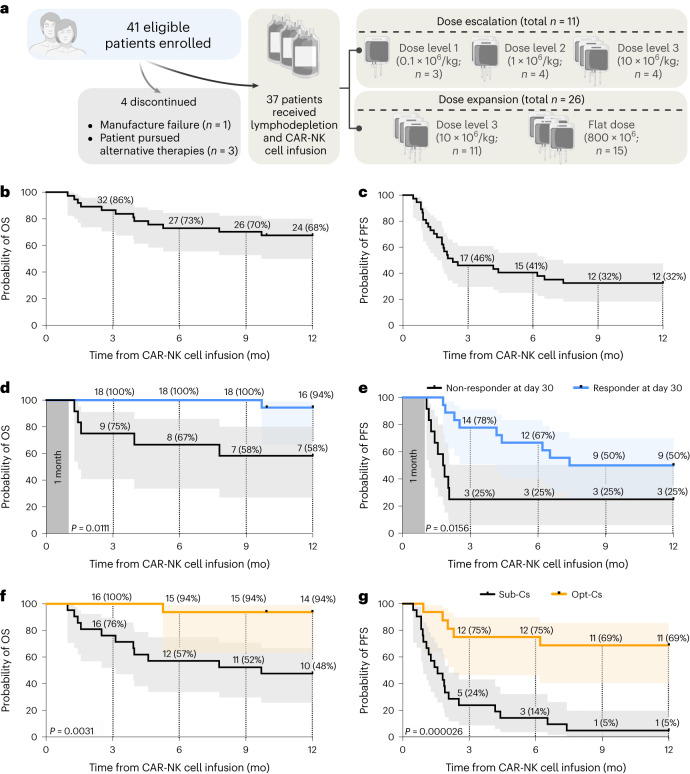
Clinical outcomes after CAR19/IL-15 NK cell therapy. **a**, Consort diagram of CAR19/IL-15 NK cell therapy (created using BioRender.com). **b**,**c**, Kaplan–Meier curves showing OS (**b**) and PFS (**c**) of patients (*n* = 37) who received CAR19/IL-15 NK cell therapy. **d**,**e**, Landmark analysis based on day 30 response evaluation for the 30 patients who remained on the study after CAR19/IL-15 NK cell therapy (*n* = 18 responders versus 12 non-responders). The Kaplan–Meier curves show the OS (**d**) and PFS (**e**) according to OR on day 30. **f**,**g**, Kaplan–Meier curves showing OS (**f**) and PFS (**g**) of patients who received CAR19/IL-15 NK cell therapy derived from Opt-Cs (*n* = 16) versus Sub-Cs (*n* = 21). Tick marks indicate the times at which data were censored for a given patient. Numbers above each line represent the number of patients at risk. Numbers in parentheses represent the probabilities of OS or PFS at a given time point; mo, months. *P* values were determined by log-rank test, and the shaded areas represent 95% CI of survival probability. Source data

Between 30 June 2017 and 27 May 2021, we conducted a phase 1/2 clinical trial at MD Anderson Cancer Center (MDACC) to assess the safety and efficacy of CAR19/IL-15 NK cells in 37 patients with CD19-positive malignancies (Fig. 1a, Table 1 and Supplementary Table 1). Patients aged 7–80 years with relapsed/refractory CD19-positive B cell malignancies, a Karnofsky performance status of >70%, an adequate organ function and no prior history of anti-CD19-directed therapy were eligible. The study had two phases, a dose-escalation phase (*n* = 11) that was previously reported^13^ and an expansion phase (*n* = 26). In the expansion phase, patients were initially treated at the 10^7^ cells per kilogram of body weight CAR19/IL-15 dose level; then the trial was amended, and the remaining 15 patients received a flat dose of 8 × 10^8^ CAR19/IL-15 to facilitate the eventual use of an off-the-shelf product (Methods). The primary objectives were safety and efficacy defined as day 30 OR. Secondary objectives included day 100 response, progression-free survival (PFS), overall survival (OS) and CAR19/IL-15 persistence.

**Table 1:** Patient, disease, donor CBU and CAR-NK characteristics

Variable	*n* (%)
Patient characteristics	
**Age**	
Median (range)	64 (26–79)
<65 years	19 (51.4)
≥65 years	18 (48.6)
**Sex**	
Male	24 (64.9)
Female	13 (35.1)
**Race**	
Caucasian	31 (83.8)
Hispanic	0 (0.0)
Black	1 (2.7)
Asian	2 (5.4)
Other	3 (8.1)
**Weight**	
Median (kg; range)	78 (47–140)
**Diagnosis**	
Indolent lymphoma^a^	6 (16.2)
CLL	6 (16.2)
CLL-RT	5 (13.5)
DLBCL	17 (45.9)
Mantle cell lymphoma	1 (2.7)
Acute lymphoblastic leukemia	1 (2.7)
Lymphoplasmacytic lymphoma	1 (2.7)
**Karnofsky (%)**	
Median (range)	90 (80–100)
**LDH before conditioning chemotherapy**	
Median (range)	259 (141–1765)
LDH > ULN	24 (64.9)
**Number of prior therapies**	
Median (range)	4 (2–10)
≥3 prior lines of therapy	31 (83.8)
**Prior stem cell transplant**	
Autologous	9 (24.3)
Allogeneic	1 (2.7)
**Disease status**	
Relapsed	11 (29.8)
Refractory to most recent therapy	16 (43.2)
History of primary refractory disease	10 (27.0)
**Disease stage**^b^	
I or II	8 (36.4)
III or IV	14 (63.6)
**CAR-NK characteristics**	
**Dose level**	
1 × 10^5^ cells/kg	3 (8.1)
1 × 10^6^ cells/kg	4 (10.8)
1 × 10^7^ cells/kg	15 (40.5)
8 × 10^8^ cells flat dose	15 (40.5)
**Fold expansion day 0–15**	
Median (range)	953.0 (14.9–7,630.0)
25% percentile	450.2
**CAR MFI**^c^	
Median (range)	12,975 (4,836–36,328)
25% percentile	9,343.5
**Transduction efficiency (%)**	
Median (range)	72.4 (22.7–91.1)
25% percentile	52.8
**CD3**^+^ **T cells infused per kg**	
Median (range)	2,000 (30–16,000)
**CBU characteristics**	
**HLA allelic match**	
0/6	5 (13.5)
1/6	9 (24.3)
2/6	2 (5.4)
3/6	2 (5.4)
4/6	19 (51.4)
**KIR ligand mismatch**	
No	21 (56.8)
Yes	16 (43.2)
**Sex**	
Male	22 (60.0)
Female	15 (40.0)
**CBU race**	
Asian	1 (2.7)
Black	1 (2.7)
Hispanic	11 (29.7)
Caucasian	23 (62.2)
Multiple	1 (2.7)
**Time from collection to freezing (h)**	
Median (range)	24.3 (13.4–42.4)
**Pre-freezing CBU viability (%)**	
Median (range)	99.0 (89.0–100)
**Days in culture**	
Median (range)	15 (15–22)
**TNC content (×10**^**7**^ **cells)**	
Median (range)	168.6 (87.3–248.8)
25% percentile	128.1
**NRBC content (×10**^**7**^ **cells)**	
Median (range)	4.8 (0.0–23.8)
75% percentile	8.0

CLL-RT, chronic lymphocytic leukemia with Richter’s transformation; LDH, lactate dehydrogenase; ULN, upper limit of normal; TNC, total nucleated cell.

^a^Four patients had follicular lymphoma and two patients had marginal zone lymphoma.

^b^Only for NHL patients.

^c^CAR MFI was determined based on expression on the total NK cell population.

### Safety

Safety was the primary objective of this trial. None of the patients developed neurotoxicity or graft-versus-host disease and only one developed CRS (grade I). Lymphodepleting chemotherapy caused reversible hematological toxicity in all patients (Table 2), and the maximum tolerated dose was not reached.

**Table 2:** List of adverse reactions

Adverse reactions	Grade			Adverse reactions	Grade		
	I–II	III	IV		I–II	III	IV
**CRS**	1	0	0	**Constitutional**			
**Graft-versus-host disease**	0	0	0	Anorexia	1	0	0
**Neurological toxicity**	0	0	0	Asthenia	12	1	0
**Hematological toxicity**				Fever/chills^a^	2	0	0
Neutropenia	3	5	29	Insomnia	2	0	0
Lymphopenia	0	0	37	Somnolence	0	1	0
Thrombocytopenia	23	4	6	**Gastrointestinal**			
Anemia	19	18	0	Diarrhea	4	0	0
Petechia/other bleeding	1	0	0	Hemorrhoids	3	0	0
**Laboratory values**				Ileus	0	2	0
Elevated creatinine	4	0	0	Mucositis	4	0	0
Elevated LFTs	9	1	0	Nausea/vomiting	8	0	0
Elevated CRP	2	0	0	**Cardiovascular**			
Elevated ferritin	5	0	0	Chest pain	2	0	0
Hyperglycemia	1	0	0	Dysrhythmia	7	0	0
Elevated LDH	3	0	0	Hypertension	1	0	0
Hypomagnesemia	1	0	0	Hypotension	5	0	0
**Respiratory**				Edema/fluid overload	2	1	0
Dyspnea	1	0	0	**Miscellaneous**			
Pleural effusion	0	1	0	Anxiety	3	0	0
Pneumonitis	1	1	0	Headache/dizziness	2	0	0
**Infection**				Muscle/bone pain	4	0	0
Bacterial infection	3	1	0	Paresthesia	4	0	0
Viral infection	4	3	0	Rash/skin discoloration	4	0	0
Febrile neutropenia^b^	0	4	0	Other	2	0	0

LFT, liver function test; CRP, C-reactive protein; LDH, lactate dehydrogenase.

The table includes all side effects from the time of infusion until day +40, irrespective of their attribution to the CAR19/IL-15 NK cell therapy. Abnormalities due to the original disease were not captured.

^a^One patient had isolated chills; one patient had fever and was classified as CRS corresponding to the reported CRS case.

^b^One patient had fever related to MRSA pneumonia; one patient had a fever that resolved within 24 h of starting antibiotics; one patient had two neutropenic fever episodes (the first episode occurred before the cells were given and the second episode was a recurrence of this fever and did not fit criteria for CRS).

### Response to therapy

**Extended Data Fig. 1:**
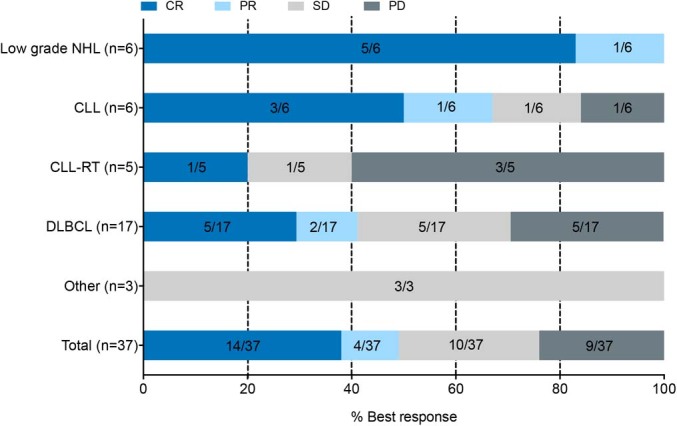
Clinical response following CAR19/IL-15 NK cell therapy according to the underlying disease. Bar graph showing the diagnosis and best response for the 37 patients treated in the study; CR: complete response; PR: partial response; SD: stable disease; PD: progressive disease; NHL: non-Hodgkin’s lymphoma; low-grade NHL: follicular lymphoma and marginal zone lymphoma; CLL: chronic lymphocytic leukemia; CLL-RT: CLL with Richter’s transformation; DLBCL: diffuse large B cell lymphoma; Other: mantle cell lymphoma (n = 1), acute lymphoblastic leukemia (n = 1), and lymphoplasmacytic lymphoma (n = 1).

A primary objective of the study was efficacy, defined as the patient being alive and in at least partial remission on day 30 after CAR-NK cell infusion. Secondary objectives included day 100 response, PFS and OS. The day 30 and day 100 OR rates including partial response (PR) and complete response (CR) for the 37 patients in the study were 48.6% (18/37; 95% confidence interval (CI) = 31.9–65.6%) for both. The day 30 and day 100 CR rates for the 37 patients were 27% (10/37; 95% CI = 13.8–44.1%) and 29.7% (11/37; 95% CI = 15.9–47.0%), respectively. The 1-year CR rate was 37.8% (14/37; 95% CI = 22.5–55.2%). The day 30 OR and 1-year CR rates for patients with low-grade non-Hodgkin lymphoma (NHL) were 100% (6/6) and 83% (5/6; *n* = 6), for chronic lymphocytic leukemia (CLL) without transformation 67% (4/6) and 50% (3/6; *n* = 6), for diffuse large B cell lymphoma (DLBCL) 41% (7/17) and 29% (5/17; *n* = 17), and for CLL with Richter’s transformation 20% (1/5) and 20% (1/5; *n* = 5), respectively (Extended Data Fig. 1 and Table 3). Figure 1b,c shows the 1-year OS and PFS for the whole group. The median time to first response was 30 d (range 30–55 d). Responses were durable; nine of the ten patients who had achieved a CR by day +30 remained in CR on day +180. Furthermore, four of eight patients who achieved a PR on day +30 eventually achieved a CR. Patients who achieved a CR by day 30 after infusion had a 70.0% (95% CI = 39.7–89.2%) probability of remaining in CR at 12 months. Post-remission therapy was permitted after the day 30 assessment only in the dose-escalation part of the study, with two patients having received a hematopoietic stem cell transplant as previously described^13^. No patients received additional therapy in the dose-expansion part of the study.

**Table 3:** Outcomes according to patient, disease, donor CBU and CAR-NK characteristics

Variable	*n* (%)	Day + 30 OR (%)*	1-year CR (%)*	1-year PFS (95% CI)^†^	1-year OS (95% CI)^†^
**Patient and disease characteristics**					
**Diagnosis**		*P* = 0.013	*P* = 0.08	*P* = 0.46	*P* = 0.03
Low-grade NHL^a^	6	6 (100)	5 (83.3)	50.0 (18.8–81.2)	100
CLL	6	4 (66.7)	3 (50.0)	33.3 (9.7–69.9)	83.3 (43.7–97.0)
CLL-RT	5	1 (20.0)	1 (20.0)	20.0 (3.6–62.5)	20.0 (3.6–62.5)
DLBCL	17	7 (41.2)	5 (29.4)	29.4 (13.2–53.2)	64.7 (41.3–82.7)
Other	3	0 (0.0)	0 (0.0)	33.3 (6.1–79.2)	66.7 (20.8–93.9)
**Age**		*P* = 0.75	*P* = 0.31	*P* = 0.59	*P* = 0.97
<65 years	19	10 (52.6)	9 (47.4)	36.8 (19.1–59.0)	68.4 (45.9–84.7)
≥65 years	18	8 (44.4)	5 (27.8)	27.8 (12.5−51.0)	66.7 (43.8–83.7)
**Sex**		*P* = 0.09	*P* = 0.17	*P* = 0.97	*P* = 0.71
Male	24	9 (37.5)	7 (29.2)	37.5 (21.1–57.3)	66.7 (46.8–82.0)
Female	13	9 (69.2)	7 (53.8)	23.1 (8.2–50.3)	69.2 (42.3–87.3)
**Karnofsky**		*P* = 0.02	*P* = 0.17	*P* = 0.87	*P* = 0.06
≤90	24	8 (33.3)	7 (29.2)	33.3 (18.0–53.2)	54.2 (35.1–72.2)
100	13	10 (76.9)	7 (53.8)	30.8 (12.7–57.7)	92.3 (66.6–98.6)
**LDH before lymphodepleting chemotherapy**		*P* = 1.0	*P* = 1.0	*P* = 0.38	*P* = 0.98
Normal	13	6 (46.2)	5 (38.5)	46.2 (23.3–70.9)	64.7 (36.1–85.6)
Elevated	24	12 (50.0)	9 (37.5)	25.0 (12.0–44.8)	69.7 (49.2–84.5)
**Number of prior therapies**		*P* = 0.41	*P* = 0.65	*P* = 0.86	*P* = 0.44
1–2	6	4 (66.7)	3 (50.0)	33.0 (9.5–69.8)	83.3 (43.7–97.0)
≥3	31	14 (45.2)	11 (35.5)	32.3 (18.6–49.9)	64.5 (46.9–78.9)
**Prior stem cell transplant**		*P* = 1.00	*P* = 1.00	*P* = 0.45	*P* = 0.36
No	27	13 (48.1)	10 (37.0)	29.6 (15.8–48.5)	63.0 (44.2–78.5)
Yes	10	5 (50.0)	4 (40.0)	40.0 (16.8–68.7)	80.0 (49.1–94.3)
**Disease status**		*P* = 0.54	*P* = 0.30	*P* = 0.58	*P* = 0.30
Relapsed	11	7 (63.6)	6 (54.5)	45.5 (21.3–72.0)	81.8 (52.4–94.8)
Refractory to last therapy	16	7 (43.8)	6 (37.5)	25.0 (10.2–49.4)	68.8 (44.4–85.9)
Primary refractory	10	4 (40.0)	2 (20.0)	30.0 (10.8–60.3)	50.0 (23.7–76.3)
**CBU characteristics**					
**HLA allelic match**^b^		*P* = 0.85	*P* = 0.95	*P* = 0.90	*P* = 0.45
0/6	5	3 (60.0)	2 (40.0)	40.0 (11.8–76.9)	60.0 (23.1–88.2)
1/6	9	5 (55.6)	4 (44.4)	33.3 (12.0–64.5)	88.9 (56.4–98.0)
2/6	2	1 (50.0)	1 (50.0)	50.0 (9.4–90.6)	50.0 (9.4–90.6)
3/6	2	0 (0.0)	0 (0.0)	50.0 (9.4–90.6)	100.0
4/6	19	9 (47.4)	7 (36.8)	26.3 (11.8–48.8)	57.9 (36.3–76.8)
**KIR ligand mismatch**		*P* = 0.51	*P* = 1.00	*P* = 0.35	*P* = 0.67
No	21	9 (42.9)	8 (38.1)	28.6 (13.8–50.1)	71.4 (49.9–86.2)
Yes	16	9 (56.3)	6 (37.5)	37.5 (18.5–61.4)	62.5 (38.6–81.5)
**KIR haplotype**		*P* = 1.0	*P* = 0.44	*P* = 0.79	*P* = 0.73
A	8	4 (50.0)	4 (50.0)	37.5 (13.7–69.4)	75.0 (40.9–92.8) 65.5
B	29	14 (48.3)	10 (34.5)	31.0 (17.2–49.2)	(47.4–80.0)
**CBU race**		*P* = 0.091	*P* = 0.035	*P* = 0.40	*P* = 0.08
Other	14	4 (28.6)	2 (14.3)	28.6 (11.7–54.7)	50.0 (26.7–73.3)
Caucasian	23	14 (60.9)	12 (52.2)	34.8 (18.9–55.1)	78.3 (58.1–90.4)
**CBU gender**		*P* = 0.18	*P* = 0.31	*P* = 0.06	*P* = 0.12
Female	15	5 (33.3)	4 (26.7)	20.0 (7.1–45.1)	53.3 (30.1–75.2)
Male	22	13 (59.1)	10 (45.5)	40.9 (23.2–61.3)	77.3 (56.7–89.9)
**Time to freezing**		*P* = 0.068	*P* = 0.017	*P* < 0.001	*P* = 0.023
>24 h	18	6 (33.3)	11 (57.9)	5.6 (1.0–25.7)	50.0 (29.0–71.0)
≤24h	19	12 (63.2)	3 (16.7)	57.9 (36.3–76.8)	84.2 (62.3–94.5)
**Pre-freezing viability**		*P* = 0.41	*P* = 0.22	*P* = 0.03	*P* = 0.39
<97%	7	2 (28.6)	1 (14.3)	0 (-)	57.1 (25.0–84.1)
≥97%	30	16 (53.3)	13 (43.3)	40.0 (24.7–57.6)	70 (52.1–83.4)
**NRBC content**		*P* = 0.019	*P* = 0.007	*P* < 0.001	*P* = 0.06
≤8 × 10^7^ cells	28	17 (60.7)	14 (50.0)	42.9 (26.5–61.1)	75.0 (56.6–87.3)
>8 × 10^7^ cells	9	1 (11.1)	0 (0.0)	0 (-)	44.4 (18.8–73.4)
**TNC content**		*P* = 0.73	*P* = 0.27	*P* = 0.57	*P* = 0.20
≤25% percentile	26	12 (46.2)	8 (30.8)	26.9 (13.7–46.1)	61.5 (42.6–77.5)
>25% percentile	11	6 (54.5)	6 (54.5)	45.5 (21.3–72.0)	81.8 (52.4–94.8)
**Optimal CBU**		*P* = 0.008	*P* = 0.002	*P* < 0.001	*P* = 0.003
no	21	6 (28.6)	3 (14.3)	4.8 (0.9–22.4)	47.6 (28.3–67.6)
Yes	16	12 (75.0)	11 (68.8)	68.8 (44.4–85.9)	93.8 (71.4–98.9)
**CAR-NK characteristics**					
**Transduction efficiency**		*P* = 0.71	*P* = 0.25	*P* = 0.17	*P* = 0.11
≤25% percentile	9	5 (55.6)	5 (55.6)	44.4 (15.8–73.1)	44.4 (18.8–73.4)
>25% percentile	28	13 (46.4)	9 (32.1)	28.6 (15.3–47.0)	75.0 (56.6–87.3)
**Fraction expansion**		*P* = 0.12	*P* = 0.43	*P* = 0.48	*P* = 0.62
≤25% percentile	9	2 (22.2)	2 (22.2)	22.2 (6.3–54.8)	61.5 (35.5–82.3)
>25% percentile	28	16 (57.1)	12 (42.9)	35.7 (20.6–54.2)	70.8 (50.8–85.1)
**Number of days in culture**		*P* = 0.75	*P* = 1.0	*P* = 0.28	*P* = 0.24
15	20	9 (45.0)	8 (40.0)	25.0 (11.2–46.9)	60.0 (38.6–78.2)
22	17	9 (52.9)	6 (35.3)	41.2 (21.7–64.0)	76.5 (52.7–90.5)
**Dose level**		*P* = 0.43	*P* = 0.21	*P* = 0.39	*P* = 0.10
1 × 10^5^ cells/kg	3	2 (66.7)	2 (66.7)	66.7 (20.8–93.9)	66.7 (20.8–93.9)
1 × 10^6^ cells/kg	4	3 (75.0)	3 (75.0)	25.0 (4.5–70.0)	100.0
1 × 10^7^ cells/kg	15	5 (33.3)	4 (26.7)	20.0 (7.1–45.1)	46.7 (24.8–69.9)
8 × 10^8^ cells flat dose	15	8 (53.3)	5 (33.3)	40.0 (19.9–64.2)	80.0 (54.9–92.9)

DLBCL, diffuse large B cell lymphoma.

^a^Low-grade lymphoma includes follicular lymphoma and marginal zone lymphoma.

^b^Number of HLA matches between the CBU and the patient at HLA loci A, B and DRβ1.

*P values were derived by two-tailed Fisher’s exact test.

^†^*P* values were derived by two-tailed log-rank test.

### Outcomes based on day + 30 response

As a post hoc analysis, we performed a landmark analysis^19,20^, excluding the 7 patients who had progressed before day 30. We classified the remaining patients according to their day 30 response. The 18 patients who had achieved an OR had significantly superior 1-year probabilities of OS (94.4% versus 58.3%, *P* = 0.01) and PFS (50.0% versus 25.0%, *P* = 0.016) compared to the 12 patients who failed to respond (Fig. 1d,e).

### CAR-NK cell expansion and persistence

**Extended Data Fig. 2:**
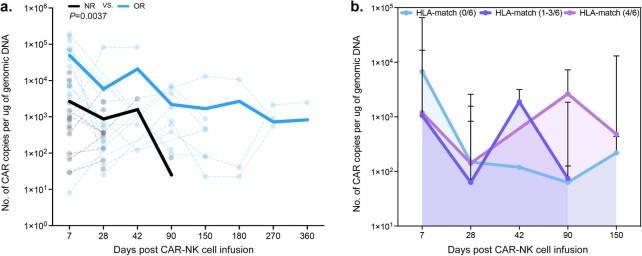
CAR-NK cell persistence in the peripheral blood (PB) of patients after CAR19/IL-15 NK-cell infusion. (**a**) Measurements of CAR-NK cells in PB samples (CAR copy number) by quantitative polymerase-chain-reaction (qPCR) in overall responders (ORs, blue; n = 18 patients) vs. non-responders (NRs, black; n = 19 patients) after treatment with CAR19/IL-15 NK cells. Each dot represents a measurement for one patient at one time point. Measurements for individual patients are connected using dashed lines. The solid lines represent the mean values for each group. (**b**) Measurements of CAR copy number by qPCR in PB samples of patients according to the degree of match at HLA-A, HLA-B and HLA-DR loci between the cord donor and the patient; HLA match 0/6 (n = 5 patients), HLA match 1-3/6 (n = 13 patients), HLA match 4/6 (n = 19 patients). Data are shown as median + 95% CI. P-values were determined by mixed-effects model with Geisser-Greenhouse correction in panel **a**. Source data

As a secondary objective of this trial, we measured CAR-NK cell persistence in serial peripheral blood (PB) samples by quantitative PCR (qPCR). Patients who achieved OR had higher levels and longer persistence of CAR-NK cells (Extended Data Fig. 2a). There was no significant difference in the persistence of CAR-NK cells according to the degree of HLA mismatch with the recipient (Extended Data Fig. 2b).

### Trogocytosis as a predictor of relapse

**Extended Data Fig. 3:**
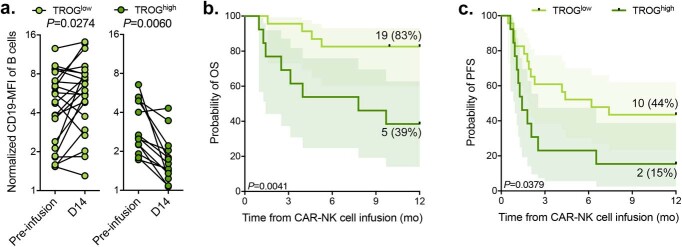
Expression of trogocytosis (TROG) antigen on CAR19 + NK cells is associated with a reduction in CD19 expression on B cells and predicts for worse outcomes after CAR19/IL-15 NK-cell treatment. (**a**) Comparison of normalized CD19-MFI expression on B cells in the PB of patients before and 14 days after CAR19/IL-15 NK cell therapy. The data are shown for TROG^low^ (n = 23 patients; left) and TROG^high^ groups (n = 13 patients; right). (**b** and **c**) Kaplan-Meier curves showing (**b**) overall survival (OS) and (**c**) progression-free survival (PFS) for patients categorized as TROG^low^ (n = 23 patients) vs. TROG^high^ (n = 13 patients); mo: months. Numbers above each line represent the number of patients at risk. Numbers in parentheses represent the probabilities of OS or PFS at a given time point. Trogocytosis data were not available for one patient. The shaded areas represent 95% confidence interval (CI) of survival probability. P-values were determined by two-tailed paired student t test in panel **a**, or by log-rank test in panels **b** and **c**. Source data

Trogocytosis has been reported to contribute to relapse following CAR therapy by transferring the target antigen from the tumor to the CAR effector cells^21,22^, leading to a reversible posttranscriptional downregulation in tumor cell antigen expression^21^. In addition, transfer of the trogocytosed (TROG) antigen from the tumor to the CAR effector cell triggers self-recognition and fratricide by sibling cells^21,22^. Thus, as a post hoc analysis, we investigated the impact of trogocytosis on outcomes by measuring CD19 levels on both CAR-NK cells and B cells in PB samples from patients after CAR-NK cell infusion. Patients were divided into two groups: TROG^high^, defined as high trogocytic CD19 (tCD19) expression on CAR-NK cells (*n* = 13 patients) and TROG^low^, defined as low/absent tCD19 expression on CAR-NK cells (*n* = 23 patients; Methods). Following CAR-NK cell infusion, we observed a downregulation in CD19 expression on B cells from patients in the TROG^high^ group, compared to those in the TROG^low^ group (Extended Data Fig. 3a). The patients with high trogocytosis had a worse 1-year OS (38.5% versus 82.6%, *P* = 0.0041), PFS (15.4% versus 43.5%, *P* = 0.0379) and CR rate (7.7% versus 56.5%, *P* = 0.005) than the 23 patients with low levels (Extended Data Fig. 3b,c).

Prior studies also point to genetic mutations resulting in total target antigen loss as an important mechanism of relapse following CAR19 T cell therapy^23,24^. Thus, we also analyzed CD19 expression in lymph node and bone marrow biopsy samples at the time of relapse for a subset of patients (*n* = 8) with available samples. Although we detected modest CD19 expression reduction in one patient, our findings did not show evidence of target antigen loss after CAR19/IL-15 NK cell therapy.

### DSAs and cytokine profile

**Extended Data Fig. 4:**
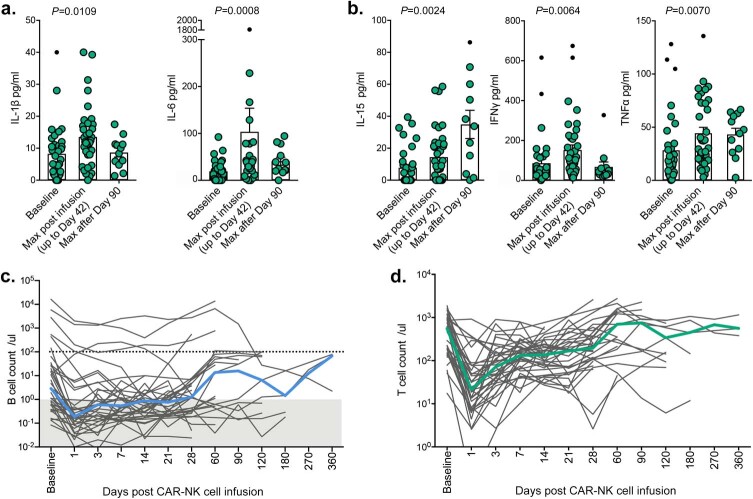
Levels of cytokines in the PB of patients following treatment with CAR19/IL-15 NK cells. Bar graphs showing the levels of cytokines (markers of cytokine release syndrome in (**a**), and effector cytokines in (**b**) in the PB of patients at baseline and the maximum values in the first 6 weeks or at 3 months or later after CAR19/IL-15 NK-cell infusion. (**a**) IL-1β (left), and IL-6 (right) (n = 33). (**b**) IL-15 (left), IFN-γ (middle), TNF-α (right) (n = 34). (**c**) Spider plot showing B cell counts calculated based on CD19+ B-cell frequencies by flow cytometry in PB samples collected from patients at baseline and at multiple timepoints post CAR-NK cell infusion (n = 37 patients). The dotted line represents the threshold for B-cell lymphopenia (<100 B-cells/μL). The shadowed area represents B-cell aplasia (<1 B-cell/μL). The solid blue line represents the mean. (**d**) Spider plot showing CD3+ T cell counts calculated based on flow cytometry in PB samples from patients at baseline and at multiple timepoints post CAR-NK cell infusion (n = 37 patients). The solid green line represents the mean. For panels **a**, **b**, each symbol represents an individual patient, data are shown as mean + s.e.m. P-values were determined by Kruskal-Wallis test in panels **a**, **b**. Each symbol represents an individual patient; outliers are identified as black dots. Source data

As part of exploratory analyses, we measured donor-specific antibodies (DSAs) and serum cytokines. Of 37 patients, only 2 developed DSAs targeting the mismatched HLA alleles on the CAR-NK cells. Neither had evidence of HLA antibodies at baseline. DSAs were detected against HLA-Cw6 at a mean fluorescence intensity (MFI) of 7,132 approximately 4 weeks after infusion for the first patient and against HLA-B44 at an MFI of 17,060 around 4 months following infusion for the second patient. Both patients achieved CR and had detectable CAR-NK cells by qPCR after the acquisition of the antibodies. In keeping with the clinical safety profile, we observed a modest elevation in IL-6, IL-1β and other cytokines over baseline in the sera of patients after infusion (Extended Data Fig. 4a,b).

### B cell aplasia

B cell aplasia is used as a surrogate for CAR19 T cell activity. As an exploratory analysis, we measured the frequencies of CD19-positive B cells in the PB of patients after CAR-NK cell treatment. At the time of enrollment, the majority of patients (31/37) had B cell lymphopenia (B cell count < 100 cells/μl) secondary to prior B cell-targeting therapies. This number further declined, with B cells becoming nearly undetectable by flow cytometry after CAR-NK cell infusion. However, over time, there was a gradual and modest increase in the B cell count for most patients (Extended Data Fig. 4c). Among patients with available immunoglobulin G (IgG) measurements, one-third had evidence of hypogammaglobulinemia (IgG < 400 mg dl^−1^) within the first 90 d following CAR-NK cell infusion.

Of note, the T cell count followed an expected trajectory, with a drop following lymphodepleting chemotherapy followed by recovery (Extended Data Fig. 4d).

### Donor CBU characteristics predict outcomes

**Extended Data Fig. 5:**
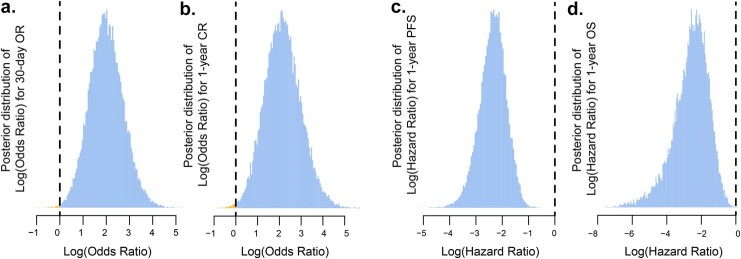
Posterior distributions of the Log (Odds Ratio) of the probability of 30-day OR, Log (Odds Ratio) of the probability of 1-year CR, Log (Hazard Ratio) of 1-year PFS and Log (Hazard Ratio) of 1-year OS for patients who received optimal cords (Opt-Cs) vs. those who did not. We computed the distribution of probabilities of the effect of receiving an Opt-C on (**a**) 30-day OR, (**b**) 1-year CR, (**c**) 1-year PFS and (**d**) 1-year OS. The area under the curve of a probability plot equals 1 and can be used to compute the probabilities of specific outcomes. For example, in panel **a**, the area to the right of Log (Odds Ratio) 2, which is about 0.50, is the probability that the Log (Odds Ratio) for the effect of Opt-C on the 30-day OR rate is larger than 2. Probability of a beneficial effect (PBE) of the variable of interest on a particular outcome is defined as the area in the distribution of probability to the right of 0. This is the probability that the rate of a favorable outcome, either (**a**) 30-day OR or (**b**) 1-year CR, is higher for patients who received Opt-C NK cells compared to patients who did not receive Opt-C NK cells. In general, a PBE near 1 implies that the variable is likely to have a beneficial effect, a PBE near 0 implies that the variable is unlikely to have a beneficial effect, and a PBE near 0.50 corresponds to no effect. For the outcomes of (**c**) 1-year PFS and (**d**) 1-year OS, PBE is represented graphically by the portion in the distribution of probabilities of Log (Hazard Ratio) to the left of 0, since it is better to have a smaller risk of death for OS, or for progression or death for PFS.

As part of our exploratory research analyses, we investigated whether CBU characteristics can influence patient outcomes (Table 3). To account for the relatively small sample size, we used Bayesian models to estimate the effect of a given covariate on a particular outcome. This effect was quantified by the probability of a beneficial effect (PBE), defined as the probability of a better outcome when the variable is present. A PBE near 0 implies a very harmful effect of the covariate, a value near 1 implies a very beneficial effect, and a PBE close to 0.50 corresponds to no effect. Among the various CBU characteristics, multivariate analyses showed that time from collection to cryopreservation ≤ 24 h (hazard ratio (HR) = 0.137, 95% credible interval (95CrI) 0.054–0.317, PBE = 1.00) and nucleated red blood cell (NRBC) content ≤ 8 × 10^7^ cells per CBU (HR = 0.119, 95CrI 0.052–0.4283, PBE = 1.00) were the only independent predictors for 1-year PFS. We defined the CBUs that fulfilled both characteristics of time from collection to cryopreservation ≤ 24 h and NRBC ≤ 8 × 10^7^ cells as optimal cords (Opt-Cs). The 16 patients who received CAR19/IL-15 NK cells from Opt-Cs had a significantly superior probability of day +30 OR (odds ratio = 7.41, 95CrI 1.86–33.11, PBE = 0.998), 1-year CR (odds ratio = 12.40, 95CrI 2.78–65.04, PBE = 1.00), 1-year PFS (HR = 0.094, 95CrI 0.032–0.239, PBE = 1.00) and 1-year OS (HR = 0.073, 95CrI 0.01–0.384, PBE = 1.00) compared to the 21 patients who received cells derived from CBUs with a high NRBC content (a surrogate for fetal hypoxia and stress)^25,26^ and/or a longer collection-to-cryopreservation time (suboptimal cords (Sub-Cs); Fig. 1f,g, Extended Data Fig. 5 and Table 3).

Next, we investigated patient, disease and donor characteristics that may influence outcomes. Multivariate regression analyses showed that, when accounting for the effects of the covariates in Table 3, receiving CAR19/IL-15 NK cells generated from an Opt-C was strongly associated with higher probabilities of day +30 OR (odds ratio = 13.05, 95CrI 1.50–137.4, PBE = 0.991), 1-year CR (odds ratio = 9.00, 95CrI 1.12–82.46, PBE = 0.981), 1-year PFS (HR = 0.041, 95CrI 0.012–0.134, PBE = 1.000) and 1-year OS (HR = 0.053, 95CrI 0.006–0.331, PBE = 1.000). Karnofsky score > 90% was associated with a higher probability of day +30 OR (odds ratio = 5.80, 95CrI 0.69–55.31, PBE = 0.946) and probability of 1-year survival (HR = 0.135, 95CrI 0.011–0.906, PBE = 0.981).

Trogocytosis was observed in a significantly greater proportion of patients treated with CAR19/IL-15 NK cells derived from Sub-Cs (TROG^high^: 60% (12/20), *P* = 0.001) compared to those from Opt-Cs (TROG^high^: 6.3% (1/16)). Trogocytosis was not included in the multivariate analysis since it was evaluated as a post hoc analysis.

### Validation of donor CBU determinants of response

**Extended Data Fig. 6:**
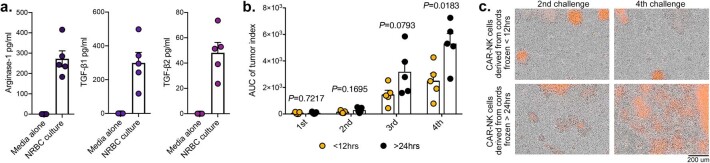
Immunosuppressive properties of NRBCs and impact of prolonged CBU collection-to-cryopreservation on CAR-NK function. (**a**) Bar graphs show levels of arginase-1 (measured by ELISA), TGF-β1 and TGF-β2 (assessed by Milliplex) released by NRBCs (500 K cells/ml) purified from CBU (n = 5 CBU donors). NRBCs were cultured for up to 72 hours and the maximum level for each analyte was plotted. Media alone was used as a negative control. (**b** and **c**) Five CBU with an NRBC count ≤8 × 10^7^ were each divided into two fractions (Fractions A and B) soon after collection. Fraction A was cryopreserved <12 hours from collection while fraction B was frozen 24-48 hours post-collection in the MD Anderson CB bank. Both fractions were then thawed at the same time and NK cells isolated, expanded, and transduced with the CAR19/IL-15 retroviral vector following our standard procedure in our GMP facility. We used the IncuCyte® imaging system to assess the cytotoxicity of CAR19/IL-15 NK cells generated from matched Fractions A and B from the same CBU (n = 5 CB donors) against Raji^mCherry^ cells (at an E:T ratio of 10:1 and adjusted for CAR transduction efficiency) in a tumor rechallenge assay. Tumor cells were added every 2-3 days for 9 days and tumor cell killing measured by the tumor cell index. (**b**) The bar plots show the area under curve (AUC) of tumor cell index, representing the tumor cell count detected by mCherry. The AUCs for 4 tumor challenges, as a measure of the anti-tumor activity of CAR19/IL-15 from Fractions A and B, are displayed (n = 5 donors per each fraction). (**c**) Representative images from the serial tumor rechallenge assay from the experiment described in panel **b**. P-values were determined by two-tailed Student’s t test in panel **b**. Each symbol represents an individual donor, data are shown as mean + s.e.m. Source data

Higher NRBCs in CBU may indicate fetal hypoxia and stress^25,26^, factors that could potentially lead to NK cell dysfunction^27,28^. Additionally, NRBCs have been shown to exert immunoregulatory function by releasing immunosuppressive factors^29–31^. In line with previous reports, we confirmed that NRBCs isolated from CBUs release high levels of arginase-1, transforming growth factor (TGF)-β1 and TGF-β2 (Extended Data Fig. 6a).

To determine the influence of time from collection to cryopreservation on NK cell function, CBUs with NRBC counts below the threshold level of ≤8 × 10^7^ were each divided into two equal fractions after collection. The first fraction (fraction A) was cryopreserved within 12 h of collection, while the second fraction (fraction B) was cryopreserved within 24–48 h of collection. The cord fractions were thawed and processed simultaneously. CAR19/IL-15 NK cells were generated using our standard protocols and their antitumor efficacy tested in a tumor rechallenge assay in vitro. CAR19/IL-15 NK cells derived from fraction A exerted significantly better long-term cytotoxicity against Raji cells than those from the paired fraction B (Extended Data Fig. 6b,c).

Collectively, these experimental data support the immunosuppressive role of higher NRBCs and the negative impact of longer collection-to-cryopreservation time on CAR-NK cell function.

### Functional interrogation of NK cells from Opt-Cs and Sub-Cs

**Fig. 2:**
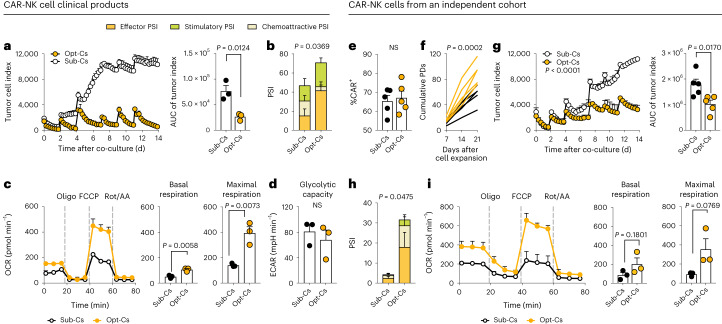
CAR19/IL-15 NK cells from Opt-Cs demonstrate superior effector function compared to those from Sub-Cs. Differences in the function of the clinical CAR19/IL-15 CBU-NK cells from Opt-Cs and Sub-Cs (**a**–**d**). Plots in **e**–**i** represent CAR19/IL-15 NK cells from an independent cohort of CBUs. **a**, Tumor rechallenge assay with CAR19/IL-15 NK cells from either Sub-Cs or Opt-Cs against Raji^mCherry^ (effector-to-target (E:T) ratio of 5:1). Tumor cells (100,000 cells) were added every 2–3 d; target killing was measured by mCherry detection. The bar graph shows the area under the curve (AUC) of tumor cell index (*n* = 3 donors per group). **b**, Bar graph showing the PSI of CAR19/IL-15 NK cells (*n* = 3 donors per group) following CD19 antigen stimulation. **c**, Oxygen consumption rate (OCR) as a surrogate for oxidative phosphorylation (OXPHOS) by Mito Stress Test of CAR19/IL-15 NK cells from Opt-Cs versus Sub-Cs (*n* = 3 donors each; left); bar graphs of basal respiration (middle) and maximal respiration (right) are also shown; Oligo, oligomycin, Rot/AA, rotenone/antimycin A. **d**, Bar graph of glycolytic capacity measured by Glycolysis Stress Test of CAR19/IL-15 NK cells from Opt-Cs versus Sub-Cs (*n* = 3 donors each); ECAR, extracellular acidification rate. **e**, Bar graph showing CAR percentage expression on NK cells derived from an independent cohort of Sub-Cs versus Opt-Cs (*n* = 5 donors per group). **f**, Cumulative population doublings (PDs) of CAR19/IL-15 NK cells (*n* = 5 per group). **g**, Tumor rechallenge assay with CAR19/IL-15 NK cells against Raji^mCherry^ (E:T ratio of 2:1). Tumor cells (100,000 cells) were added every 2–3 d; tumor killing was measured as in **a**. The bar graph shows the AUC of tumor cell index (*n* = 5 donors per group). **h**, Bar graph showing the PSI of CAR19/IL-15 NK cells after CD19 antigen stimulation (*n* = 3 donors per group). **i**, OCR as a surrogate for OXPHOS of CAR19/IL-15 NK cells from Opt-Cs versus Sub-Cs (*n* = 3 donors each) by Mito Stress Test (left); bar graphs of basal OCR (middle) and maximal OCR (right) are also shown. *P* values were determined by two-tailed Student’s *t*-test (**a**, **c**, **d**, **e**, **g** and **i**), or two-tailed one-way analysis of variance (ANOVA; **b**, **f** and **h**). Each symbol represents an individual sample, and data are shown as the mean + s.e.m. NS, not significant. Source data

**Extended Data Fig. 7:**
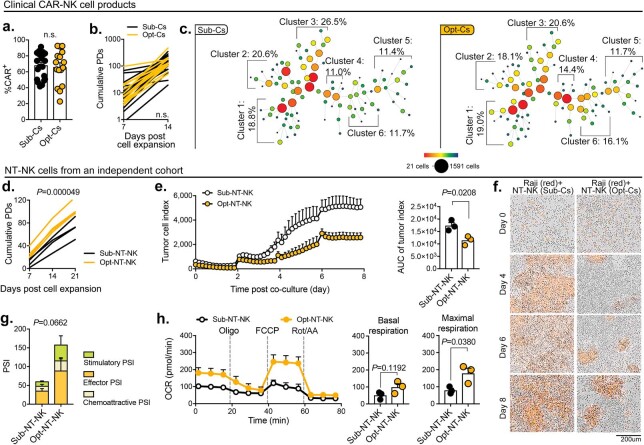
Characterization of expanded NK cells from Opt-Cs and Sub-Cs. Clinical CAR19/IL-15 NK cells from Opt-Cs and Sub-Cs were used in panels **a**-**c**. Expanded NT-NK cells from an independent cohort of CBUs were used in panels **d-f**. (**a**) Bar graph showing the percentage of CAR expression in clinical CAR19/IL-15 NK cells from Sub-Cs (n = 21 donors) or Opt-Cs (n = 16 donors). (**b**) Cumulative population doublings (PDs) for CAR-NK cells from Sub-Cs (n = 21 donors) or Opt-Cs (n = 16 donors) expanded with K562-based feeder cells and IL-2 (200 U/mL). (**c**) SPADE analysis of CyTOF data showing the phenotype of the CAR19/IL-15 NK cells (expanded for 14–21 days) from Sub-Cs (n = 17 donors) or Opt-Cs (n = 8 donors). Frequencies of each cluster (1–6) are indicated; size and color of nodes represent numbers of clustered cells. (**d**) Cumulative PDs of NT-NK cells derived from an independent cohort of Sub-Cs vs. Opt-Cs (n = 4 donors per group). (**e**) Tumor rechallenge assay where NT-NK cells were rechallenged with Raji^mCherry^ at 5:1(E:T ratio). Tumor cells (16,700 cells) were added every 2 days for 8 days and tumor cell killing was measured by the tumor cell index. The bar plots show the AUC of tumor cell index (n = 3 donors per group). (**f**) Representative images from the serial tumor rechallenge assay from the experiment described in panel **e**. (**g**) Bar graph showing the PSI of NT-NK cells secreting different cytokines after Fc-CD16 stimulation (n = 3 per group). (**h**) OCR as a surrogate for oxidative phosphorylation (OXPHOS) was performed on NT-NK cells that were expanded from a subset of Sub-Cs and Opt-Cs (n = 3 donors each). The results of the mito stress test (left), and the bar graphs of basal respiration (middle) and maximal respiration (right) are presented; Oligo: Oligomycin, Rot/AA: Rotenone/Antimycin A. P-values were determined by two-tailed Student’s t test in panels **a,e,h**, or two-tailed one-way ANOVA in panels **b,d,g**. Each symbol represents an individual donor, data are shown as mean + s.e.m. Source data

To determine the underlying mechanisms for the differences in therapeutic efficacy based on the CBU quality, we first compared CAR expression, in vitro proliferation and phenotype of the CAR19/IL-15 NK cells from the infused products. These parameters were not significantly different between NK cells from Opt-Cs and Sub-Cs (Extended Data Fig. 7a–c). An in vitro long-term tumor rechallenge assay showed that while CAR19/IL-15 NK cells from both Opt-Cs and Sub-Cs were equally effective at eliminating Raji cells (CD19 positive) after a single tumor challenge (Fig. 2a), CAR19/IL-15 NK cells from Sub-Cs lost their ability to control tumor growth upon rechallenge despite excellent viability.

Polyfunctionality and metabolic fitness are important determinants of effective antitumor NK cell responses^32,33^. Single-cell IsoPlexis analysis showed that CAR19/IL-15 NK cells from Opt-Cs had a significantly higher polyfunctional strength index (PSI) response to CD19 antigen stimulation compared to Sub-Cs (Fig. 2b). Analysis of mitochondrial metabolism and glycolytic activity showed higher oxidative phosphorylation in CAR19/IL-15 NK cells from Opt-Cs compared to Sub-Cs (Fig. 2c) with no difference in their glycolytic capacity (Fig. 2d), pointing to higher mitochondrial fitness.

To validate the findings observed with our clinical CAR19/IL-15 NK cell products, we selected 12 additional CBUs from our cord bank to generate CAR-NK cells. We confirmed that CAR-NK and non-transduced (NT)-NK cells from Opt-Cs had superior long-term cytotoxicity against Raji tumor rechallenges, while those from Sub-Cs rapidly lost their ability to control the tumor (Fig. 2e–g and Extended Data Fig. 7d–f). We also confirmed the superior PSI and mitochondrial fitness of Opt-Cs for both CAR-NK cells (Fig. 2h,i) and NT-NK cells (Extended Data Fig. 7g,h). Together, these data support the notion that the superior effector function of NK cells from Opt-Cs is not induced or mediated by CAR19/IL-15 expression.

### Multi-omic profiling of NK cells from Opt-Cs and Sub-Cs

**Fig. 3:**
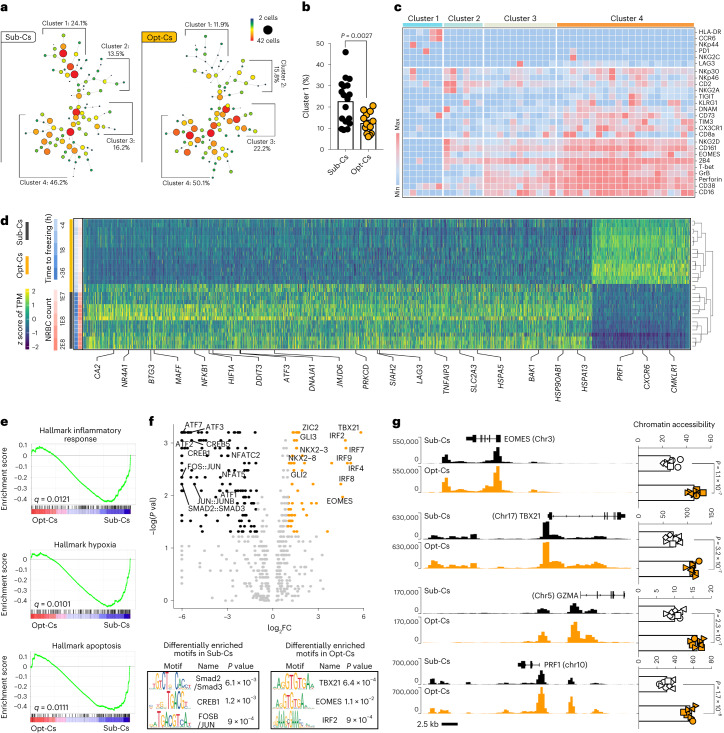
Unmanipulated NK cells from CBMCs of Opt-Cs and Sub-Cs display distinct signatures. Phenotype of unmanipulated NK cells in cryopreserved CBMCs from each of the cords used to manufacture the clinical CAR19/IL-15 NK cell product (**a**–**c**). For experiments in **d**–**g**, we used an independent cohort of CBUs. **a**, Phenotype of unmanipulated NK cells in CBMCs of Sub-Cs (*n* = 18 donors) versus Opt-Cs (*n* = 13 donors) by CyTOF. Only samples with viable CBMCs > 1,500 cells were analyzed. Frequencies of each cluster (1–4) are indicated; size and color of nodes within each cluster represent numbers of clustered cells. **b**, Bar graph shows the NK cell percentage within cluster 1 for Sub-Cs versus Opt-Cs. **c**, Heat map of marker expression within the main subclusters of clusters 1–4. Each column represents a major node within the SPADE tree clusters. The major nodes are representative of the majority of cells across all conditions. The expression level for each marker is represented from blue (low) to red (high). **d**, Heat map of differentially expressed genes (adjusted *P* value < 0.1 and absolute log_2_ fold change (FC) > 1.5) in unmanipulated NK cells from CBMCs of Opt-Cs (*n* = 18 samples) versus Sub-Cs (*n* = 14 samples). TPM, transcript per million. **e**, GSEA enrichment plots show differentially regulated pathways for NK cells from Opt-Cs versus Sub-Cs. **f**, Volcano plot (top) showing the log_2_ fold change (log_2_FC) in TF activity levels between NK cells from Sub-Cs (*n* = 9 samples, black) and Opt-Cs (*n* = 8 samples, yellow). Log plots (bottom) showing the top TF-binding motifs enriched in the preferentially open chromatin regions of unmanipulated NK cells from Opt-Cs and Sub-Cs (by HOMER). **g**, ATAC-seq tracks for selected genes in NK cells from Sub-Cs (*n* = 8 samples; top, black) versus Opt-Cs (*n* = 8 samples; bottom, yellow). Box plots comparing the gene-level accessibility score between the two groups. *P* values were determined by two-tailed Student’s *t*-test in **b** and **g**, a two-tailed Wilcoxon rank-sum test in the volcano plot and a one-tailed binomial test for motif enrichment analysis in **f**. *q* values were determined by two-tailed two-sample *t*-test with false discovery rate correction for multiple testing in **e**. Data are shown as the mean + s.e.m. Source data

There were no substantial phenotypic differences in the expanded CAR19/IL-15 NK cell products generated from Opt-Cs versus Sub-Cs (Extended Data Fig. 7c). We posited that ex vivo expansion could mask differences in the underlying phenotype of NK cells. Thus, we next examined the immune composition and the phenotype of unmanipulated NK cells in the cryopreserved cord blood mononuclear cells (CBMCs) stored in our cord bank from the cords used to manufacture the clinical CAR19/IL-15 NK cell products. There were no significant differences in the frequencies of immune subsets in the CBMCs from Opt-Cs versus Sub-Cs (Supplementary Fig. 1a,b). Cytometry by time-of-flight (CyTOF) and built-in spanning-tree progression analysis of density-normalized events (SPADE) analysis of CD45^+^CD56^+^CD3^−^ NK cells (gating strategy is shown in Supplementary Fig. 1c) revealed four main clusters (clusters 1–4; Fig. 3a). NK cells from Sub-Cs were present at higher frequencies in cluster 1, while those from Opt-Cs were overrepresented in clusters 3 and 4 (Fig. 3a,b). Clusters 3 and 4 were enriched in NK cells with a highly functional phenotype, defined by the coexpression of multiple activating receptors (NKG2D, CD16 and 2B4), transcription factors (TFs) important for NK cell activity (T-bet and EOMES), and cytotoxic granules (perforin (PFN) and granzyme A (GZMA)), while NK cells in cluster 1 did not express these functional/maturation markers (Fig. 3c). Differences in the phenotype of NK cells in CBMCs from Opt-Cs versus Sub-Cs were validated in a second set of 12 CBUs from our cord bank (Supplementary Fig. 2a–c).

**Extended Data Fig. 8:**
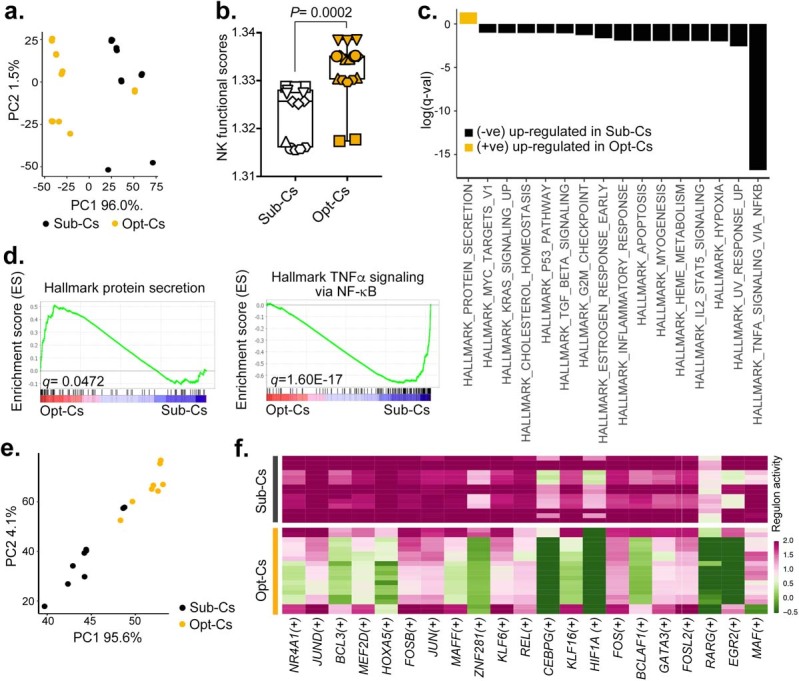
Unmanipulated NK cells from Opt-Cs and Sub-Cs are characterized by unique transcriptomic and epigenetic signatures. (**a**) PCA plot based on the top five thousand variably expressed genes, showing separation of Opt-Cs (n = 18 samples) and Sub-Cs (n = 14 samples). (**b**) Box plots showing the NK functional scores for NK cells from CBMCs of Sub-Cs (n = 13 samples) vs. Opt-Cs (n = 18 samples). Data are represented as median (min-max). (**c**) Bar graph of pathway enrichment analysis. Significantly differentially regulated pathways were identified by GSEA (q < 0.1). Positive values indicate upregulation in Opt-Cs and negative values indicate upregulation in Sub-Cs. (**d**) Enrichment plots for selected pathways identified to be differentially regulated using GSEA of NK cells from CBMCs of Opt-Cs relative to Sub-Cs. **(e)** PCA plot based on the 128,972 variably accessible peaks, showing separation of Opt-Cs (n = 8 samples) and Sub-Cs (n = 9 samples). (**f**) Heatmap showing regulon activity AUC scores of differentially active regulons (see Methods, adjusted P-value < 0.01) between Opt-C (n = 18 samples) and Sub-Cs (n = 14 samples). The AUC scores are scaled and indicated by the color intensity. P-values were determined by two-tailed Student’s t test in **b**. q-values were determined by two-tailed two-sample t test with FDR correction for multiple testing in panels **c**, **d**. P-value is determined by a two-tailed Student’s t test with Bonferroni correction in **f**. Data are shown as median with range of minimum to maximum in **b**. Source data

To investigate differences in NK cells from Opt-Cs and Sub-Cs at the transcriptomic level, we performed bulk RNA sequencing (RNA-seq) on unmanipulated NK cells purified from CBMCs of an independent set of nine CBUs. Principal component analysis (PCA) resolved samples based on the optimal/suboptimal status (Extended Data Fig. 8a), indicating their distinct transcriptomic landscapes. Analysis of differentially expressed genes in NK cells revealed important differences between the two groups (Fig. 3d). Opt-C NK cells were characterized by higher expression of effector genes like *PRF1* and higher coordinated expression of NK functional genes (Methods and Extended Data Fig. 8b) and chemokine signaling (*CXCR6* and *CMKLR1*), while Sub-C NK cells had upregulation of genes associated with hypoxia (*HIF1A*, *MAFF*, *JMJD6*, *DDIT3* and *SIAH2*), stress (*NR4A1*, *DNAJA1*, *BAK1*, *ATF3* and *NFKB1*) and immunosuppression (*IL10* and *LAG3;* Fig. 3d). Notably, genes encoding stress-related heat shock proteins such as *HSP90AB1*, *HSPA5*, *HSPA13* and *DNAJA1* were enriched in Sub-Cs compared to Opt-Cs. This pattern mirrors the stress response observed in T cells in the context of immunotherapy resistance^34^ and the poor cytotoxicity seen in tumor-associated NK cells in a recent pan-cancer single-cell atlas of human NK cells^28^.

Similarly, gene-set enrichment analysis (GSEA) revealed activation of pathways related to protein secretion in Opt-C NK cells, while pathways related to inflammation, hypoxia, apoptosis, tumor necrosis factor (TNFA) signaling via nuclear factor-kB response and DNA damage were activated in Sub-C NK cells (Fig. 3e and Extended Data Fig. 8c,d). The distinctive hypoxia signature observed in NK cells from Sub-Cs is consistent with the higher NRBC content (indicative of fetal hypoxia and stress)^25,26^ found in these cords.

To understand differences in NK cells at the epigenetic level, we performed the assay for transposase-accessible chromatin with sequencing (ATAC-seq) on ex vivo-purified unmanipulated NK cells from Opt-Cs and Sub-Cs. PCA of chromatin-accessible regions (ChARs) showed clear separation between the two groups (Extended Data Fig. 8e). We identified 13,729 differential ChARs between the two groups (logFC > 0.5, *P* < 0.05). Differential motif enrichment analysis revealed NK cells from Opt-Cs to have enrichment in motifs corresponding to TFs associated with NK effector function, for example, the interferon regulatory factor (IRF) family (IRF4, IRF7, IRF8, IRF9, IRF2 and IRF3), T-box (TBX21) and EOMES (Fig. 3f). Consistent with these findings and congruent with the activated state shown at the proteomic and transcriptomic levels, ATAC-seq track analysis revealed significantly greater accessibility at the transcription start sites and promoter regions of genes related to NK effector function such as *PRF1*, *GZMA*, *EOMES* and *TBX21* in Opt-Cs (Fig. 3g), supporting an epigenetic state poised toward increased effector function. In contrast, the motifs that were enriched in Sub-C NK cells corresponded to TFs that regulate cellular responses to stress and inflammation and that have been linked to immune dysfunction such as the AP-1 complex family (*FOS*, *JUN*, *JUNB* and *FOSL1*)^35,36^.

To integrate and validate our ATAC-seq data findings with gene expression from RNA-seq data on the same samples, we utilized the Python implementation of the SCENIC workflow (pySCENIC) to predict key regulons, score their activities and identify differentially active regulons in NK cells from Opt-Cs and Sub-Cs. We found several consistent trends between the RNA-seq and ATAC-seq analyses. Specifically, the activity of the *HIF1A* regulon, a hypoxia-induced master regulator of the cellular response to hypoxia, was significantly higher in NK cells from Sub-Cs than Opt-Cs (adjusted *P* value < 0.01), suggesting that these cells may have been exposed to hypoxic conditions (as also indicated by the higher NRBC content of the cords). In addition, several members of the AP-1 complex (*JUND*, *FOSB*, *FOS*, *JUN* and *FOSL2*) were significantly more active in Sub-C NK cells (adjusted *P* value < 0.01), consistent with the role of AP-1 in regulating cellular responses to stress and inflammation (Extended Data Fig. 8f). In summary, we observed consistent biological differences at the proteomic, transcriptomic and epigenetic levels in NK cells from the two CBU groups that may account for the superior clinical activity of CAR-NK cells generated from Opt-Cs.

### In vivo efficacy of CAR-NK cells from Opt-Cs and Sub-Cs

To investigate the in vivo antitumor function of CAR-NK cells generated from Opt-Cs versus Sub-Cs, we used three different CAR constructs and three different preclinical tumor models. Each experiment was performed with a different set of CBUs that were distinct from those used in our clinical trial or in the validation studies described above.

**Extended Data Fig. 9:**
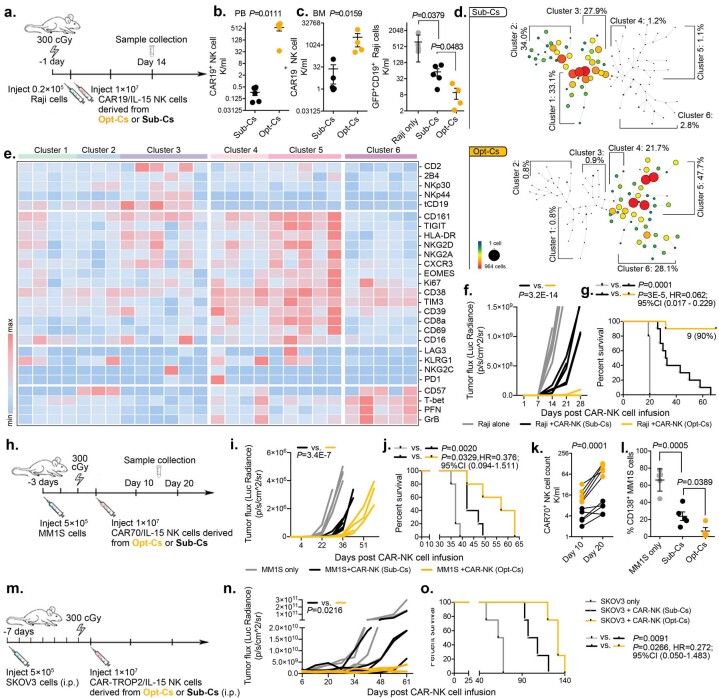
CAR-NK cells from Opt-Cs show enhanced anti-tumor activity *in vivo*. (**a**) Schema of the mouse model of Raji tumor. Mice received a single CAR19/IL-15 NK-cell infusion. For panels **b-e**, mice were sacrificed 14 days post-NK infusion. (**b**) Absolute CAR19 NK cell counts in PB 10 days post-NK cell injection (Sub-Cs: n = 5 mice; Opt-Cs: n = 4 mice). (**c**) The animals were sacrificed at day 14 after CAR19/IL-15 NK-cell injection. Absolute numbers of CAR19/IL-15 NK (left) and Raji (right) cells in BM (Raji only: n = 4 mice; Sub-Cs: n = 5 mice; Opt-Cs: n = 4 mice). (**d**) SPADE analysis showing the phenotype of live hCD45^+^CD56^+^CD3^−^CAR19/IL-15 NK cells from Sub-Cs (n = 5 mice) or Opt-Cs (n = 3 mice) from BM samples. (**e**) Heatmap representing the expression (low=blue; high=red) of NK markers within the main sub-clusters (Clusters 1–6). Each column represents a major node which is representative of the majority of cells across all conditions; tCD19: trogocytic CD19. (**f-g**) A second Raji model experiment where mice received a single infusion of CAR19/IL-15 NK-cell and were followed for tumor growth (by weekly BLI) and survival. Each line refers to an individual mouse (n = 5 mice/group). (**g**) Kaplan-Meier survival curves; data were pooled from two independent experiments. (**h**) Schema of the CD70 + MM1S tumor model. Mice received a single CAR70/IL-15 NK-cell infusion. (**i**) Plot showing MM1S tumor burden. Each line refers to an individual mouse (n = 5 mice/group). (**j**) Kaplan-Meier survival curves. (**k**) CAR70/IL-15 NK-cell count in PB at days 10 and 20 after CAR70/IL-15 NK-cell infusion. (**l**) Frequencies of CD138^+^ MM1S cells in the BM of mice collected at sacrifice. (**m**) Schema of the TROP2 + SKOV3 tumor model. Mice received a single CAR-TROP2/IL-15 NK-cell injection. (**n**) Plot showing SKOV3 tumor burden. Each line refers to an individual mouse (n = 4 mice/group). (**o**) Kaplan-Meier survival curves. P-values were determined by two-tailed two-way ANOVA in panels **b, i.k, n**, log-rank test in panels **c**, **j, o**, two-tailed Student’s t test in panels **d, e**, or two-tailed one-way ANOVA in panels **e,l**. Data shown in panels **d, e, k, l** were analyzed by flow cytometry and shown as mean ± s.e.m. Each symbol represents an individual mouse sample. Source data

First, we compared the in vivo proliferation of CAR19/IL-15 NK cells based on cord quality in a mouse model of Raji tumors. Mice were injected with Raji tumors and received CAR19/IL-15 NK cells that were generated from either an Opt-C or a Sub-C. Mice were euthanized 2 weeks later, and their blood and tissues collected for CyTOF analysis (Extended Data Fig. 9a). We observed significantly higher frequencies of circulating CAR19 + NK cells and lower tumor burden in the bone marrow of animals treated with CAR19/IL-15 NK cells from Opt-Cs compared to those from Sub-Cs (Extended Data Fig. 9b,c and Supplementary Fig. 3a). SPADE analysis segregated NK cells into six clusters, with CAR19/IL-15 NK cells from Opt-Cs dominating clusters 4–6 and those from Sub-Cs preferentially located in clusters 1–3 (Extended Data Fig. 9d). CAR19/IL-15 NK cells from Opt-Cs had significantly higher expression of TFs (EOMES and T-bet), cytolytic proteins (PFN and GrB), as well as upregulation of activating receptors (NKG2D), and lower levels of TROG-antigen acquisition (tCD19; Extended Data Fig. 9e and Supplementary Fig. 3b). In a second experiment, we studied the impact of cord quality on in vivo antitumor control and survival. CAR19/IL-15 NK cells from Opt-Cs had significantly better antitumor activity and resulted in superior survival when compared to the Sub-C CAR19/IL-15 NK cell group (Extended Data Fig. 9f,g and Supplementary Fig. 3c). We also investigated the validity of our results in a multiple myeloma mouse model of MM1S treated with anti-CD70-CAR/IL-15 (CAR70/IL-15) NK cells. CAR70/IL-15 NK cells from Opt-Cs resulted in significantly better tumor control, in vivo proliferation and superior survival compared to their Sub-C counterparts (Extended Data Fig. 9h–l and Supplementary Fig. 4a,b). Finally, in a solid tumor model of SKOV3 ovarian cancer treated with a single infusion of anti-TROP2-CAR/IL-15 NK cells (CAR-TROP2/IL-15), CAR-NK cells from Opt-Cs resulted in superior antitumor control and survival compared to those from Sub-Cs (Extended Data Fig. 9m–o and Supplementary Fig. 4c). Together, these results provide experimental evidence that CAR-NK cells generated from Opt-Cs mediate a stronger antitumor response, associated with significantly better proliferation and persistence in vivo.

## Discussion

Here, we present the final results of a first-in-human phase 1/2 study of CBU-derived engineered NK cells expressing an anti-CD19 CAR, a cytokine (IL-15) and the iC9 safety switch in 37 heavily pretreated patients with relapsed or refractory B cell malignancies. Responses were rapid and observed at all dose levels: 100% of patients with low-grade NHL, 67% of patients with CLL without transformation and 41% of patients with DLBCL achieved an OR. Most responses were CRs, with 1-year cumulative CR rates of 83%, 50% and 29% for patients with NHL, CLL and DLBCL, respectively.

CAR19/IL-15 NK cells were manufactured directly from banked CBUs, eliminating the need for leukapheresis. This makes the direct comparison of our results with those reported with autologous CAR19 T cells difficult, as most CAR-T cell studies report analysis of outcomes only for those patients who received the cells (modified intention to treat) and not from the initial screening (intention to treat). Time to treatment is an important prognostic factor in DLBCL^37^; indeed, those patients able to wait for cell manufacturing have naturally less aggressive disease. In our study, the CR rate for patients with DLBCL was 29%, which appears lower than that reported for autologous CAR19 T cells (40–64%)^38^. When the data with autologous CAR-T cells were analyzed on an intention-to-treat basis, the CR rate reported for DLBCL patients was 34% (95% CI = 27–42%)^3^, which is very similar to our results when analyzed on an intention-to-treat basis, namely 27.8% (95% CI = 10–53%). In contrast, our results for patients with more indolent disease such as low-grade NHL or CLL compared favorably with those for autologous CAR-T cells, where 71–74% of patients with indolent NHL^39,40^ and 28–45% of patients with CLL^41,42^ achieved CR.

During the phase 1 portion of the trial, two patients who had achieved a CR received a stem cell transplant. In the dose-expansion phase, post-remission therapy was not administered. Responses were durable, with a 70% probability of remaining in CR at 1 year for those patients achieving early remission. Similar results have been reported for patients with lymphoid malignancies receiving autologous CAR19 T cells^38,43^.

The use of allogeneic immune cells from healthy donors offers several advantages over autologous patient-derived cells including generation of multiple therapeutic cell doses from a single donor that could be cryopreserved for off-the-shelf use, making the allogeneic products cost effective, readily available and with the potential for a consistent and high-quality treatment. The importance of the quality of the starting material for manufacturing is very well illustrated for autologous CAR-T cell therapies, where patient baseline T cell characteristics such as polyfunctionality, increased stemness and decreased exhaustion features predict for CAR-T cell proliferation, persistence and therapeutic response^14,44–46^. However, it is important to note that even with healthy donors, there is heterogeneity with regards to their natural immunological host defenses. Indeed, in an 11-year follow-up study of >3,600 healthy donors, large variations in immune cytotoxic activity were observed among individuals. Notably, higher NK cell cytotoxicity was associated with reduced cancer risk, while lower activity was associated with increased risk^47^.

In our study, donor-related factors such as the NRBC content and the time from collection to cryopreservation were the main predictors for outcome, defining the concept of the optimal CBU. This stresses the importance of identifying donor-specific predictors of response after allogeneic cell therapy, especially since one donor may be used to treat hundreds if not thousands of patients. Such biomarkers may be relevant to cell products beyond NK cells. We have extensively validated our selection criteria using multiple experimental models. First, we measured the in vitro cytotoxicity of the CAR19/IL-15 NK cells infused to patients against CD19-expressing tumor cells and showed that CAR-NK cells from Opt-Cs had greater long-term cytotoxicity against multiple tumor rechallenges, associated with greater metabolic fitness and polyfunctionality compared to Sub-Cs. Second, we selected a different set of CBUs from our cord bank and confirmed that NK cells from Opt-Cs had greater long-term cytotoxicity and greater metabolic fitness and polyfunctionality. This was independent of whether the NK cells were transduced with CAR19/IL-15 or not, indicating that this is a NK cell-intrinsic phenomenon and not driven by the CAR. Third, we developed three different tumor mouse models; namely, Raji lymphoma treated with CAR19/IL-15 NK cells; MM1S multiple myeloma treated with CAR70/IL-15 NK cells and an ovarian SKOV3 cancer model treated with CAR-TROP2/IL-15 NK cells. For each in vivo experiment, we used a new set of Opt-Cs and Sub-Cs for CAR-NK cell generation. In each disease model, CAR-NK cells generated from Opt-Cs had better in vivo proliferation and resulted in superior tumor control. This supports the validity of our selection criteria, irrespective of the CAR or the disease model being studied. Therefore, we have implemented these criteria for donor selection in our ongoing and future clinical trials with CBU-derived NK cells.

We investigated the biological mechanisms underlying the CBU-derived NK cell variability. We did not find notable phenotypic differences in the infused CAR-NK cells; however, there were notable differences in the phenotype of the unmanipulated NK cells in the CBUs before expansion. NK cells from Opt-Cs were enriched in a population of cells with a functional phenotype, characterized by expression of activating receptors, TFs such as EOMES and T-bet and cytotoxic granules. At the transcriptomic level, and in keeping with the CyTOF analysis, NK cells from Opt-Cs had a higher functional score, while those from Sub-Cs had a signature of hypoxia likely induced by fetal hypoxia as suggested by the higher NRBC^25,26,48^ count and cellular stress possibly induced by longer time from collection to cryopreservation. Similarly, chromatin accessibility analysis by ATAC-seq revealed global differences between the two groups, with TFs associated with effector function and IRFs being more abundant in NK cells from Opt-Cs, while those associated with hypoxia (HIF1α) and cellular response to stress and inflammation^49^, such as members of the AP-1 complex, were more abundant in Sub-Cs^35,36^. Our data also suggest a degree of epigenetic scarring in NK cells from Sub-Cs as their functional impairment was not reversible by ex vivo expansion and activation, despite recovery of their phenotype.

Our study has some limitations. The selection criteria for Opt-Cs were derived from a relatively small sample size from a single CB bank. This introduces a limitation in the generalizability of the findings, highlighting the need for validation in a larger clinical cohort and with CBUs sourced from different banks. Furthermore, our data require validation in a multicenter prospective clinical trial.

In conclusion, we show that CAR19/IL-15 CBU-NK cells have a similar efficacy profile to autologous CAR19 T cells. The safety profile, however, is very different as CAR19/IL-15 CBU-NK cells are not associated with notable CRS or neurotoxicity. Moreover, our findings underscore the importance of defining the criteria for the selection of an allogeneic donor for CAR-NK cell production and identifying donor-specific predictors of response.

## Methods

### Clinical trial design

We conducted a phase 1/2 clinical trial to assess the safety and efficacy of escalating doses of CAR19/IL-15 CBU-NK cells for patients with relapsed/refractory CD19-positive malignancies. Patients were treated between June 2017 and May 2021. The first patient was enrolled on 30 June 2017 and the last patient was enrolled on 27 May 2021. Patients aged 7–80 years with relapsed/refractory CD19-positive B cell malignancies, a Karnofsky performance status of >70% and an adequate organ function were eligible. Patients must have been at least 3 weeks from the last cytotoxic chemotherapy or at least 3 d from tyrosine kinase inhibitors or other targeted therapies. Exclusion criteria included: (1) pregnancy, (2) positive serology for HIV, (3) uncontrolled infections, (4) grade III or higher toxicities from prior therapies, (5) active neurological disorders, and (6) receipt of concomitant investigational therapies. Prior CD19 targeting therapy was an exclusion criterion for the second phase of the study. The study had two phases: a dose-escalation phase and an expansion phase. The dose-escalation phase 1 (*n* = 11) was previously reported^13^. Patients received lymphodepleting chemotherapy with fludarabine 30 mg/m^2^ and cyclophosphamide 300 mg/m^2^ daily for 3 consecutive days followed by the infusion of CAR19/IL-15 CBU-NK cells at escalating doses of 10^5^ cells per kilogram of body weight, 10^6^ cells per kilogram of body weight and 10^7^ cells per kilogram of body weight. The dose was escalated using the EffTox design (see below). In the expansion phase (*n* = 26), patients were treated at the 10^7^ cells per kilogram of body weight CAR19/IL-15 CBU-NK dose level. Then the trial was amended to include a second expansion cohort where patients received a single flat dose of 8 × 10^8^ CAR19/IL-15 CBU-NK cells (the equivalent of 10^7^ cells per kilogram of body weight for an 80-kg person). The first nine patients in the phase 1 portion of the study received a CAR-NK product that was partially matched with the recipient (4/6 HLA molecules: HLA-A, HLA-B and DRβ1); the protocol was then amended to permit selection of cords with no consideration for HLA matching. The HLA mismatches were bidirectional for all patients. Table 1 shows the patient and cell product characteristics. No formal sample size computation was performed. Instead, patients were enrolled following a Bayesian EffTox dosing that included two Bayesian adaptive rules, taking into account both efficacy and toxicity outcomes. Our study protocol specified a follow-up duration of 12 months, after which the study concluded. The median follow-up time for alive patients on the study was 12 months (10–12 months). One patient was lost to follow-up at 10 months.

The study was approved by the institutional review board and conducted according to the Declaration of Helsinki. The study was overseen by the External Data Safety Monitoring Board of MDACC. Written informed consent was obtained from each patient. The trial is registered on ClinicalTrials.gov (NCT03056339).

### Safety and toxicity monitoring

The method of Thall et al. was used to determine the maximum tolerated dose and to construct a stopping bound for toxicity^50^. A dose level is considered too toxic if the maximum upper limit on probability of dose-limiting toxicity was 0.40. The following events were considered as dose-limiting toxicity: grade III or IV graft-versus-host disease within 8 weeks of NK cell infusion, CRS within 2 weeks of NK cell infusion requiring transfer to intensive care, grade IV NK cell infusion related toxicity, grades III–V allergic reactions related to study cell infusion, grades III–V organ toxicity (cardiac, dermatologic, gastrointestinal, hepatic, pulmonary, renal/genitourinary or neurologic) not preexisting or due to the underlying malignancy or due to lymphodepleting chemotherapy or treatment-related death within 8 weeks of the study cell infusion.

### Clinical trial amendments and patient enrollment

Between June 2017 and May 2021, 48 consecutives patients were enrolled in the protocol (11 were screen failures and 37 received the therapy). Patients were enrolled sequentially with a staggering interval of 14 d from the day of CAR-NK infusion to the start of the preparative regimen for the next patient within each cohort, as well as a 2-week interval as the dose was escalated to the next level. In March 2019, we considered the dose-finding portion of the study to be complete and the protocol was amended (amendment 16) to allow for the enrollment of additional patients at the 10^7^ cells per kilogram of body weight dose. In April 2020, in preparation for the introduction of a frozen product (which was not done in this trial), we changed the dose of 10^7^ cells per kilogram of body weight dose to a flat dose of 8 × 10^8^ cells (amendment 22). The last version of the protocol was version 25. The data cutoff for this report was July 2022, at which point all patients had completed the 1-year follow-up.

### Clinical responses

Clinical responses to therapy for CLL and NHL were based on the Lugano and iWCLL 2018 criteria, respectively^51,52^. OR represents the combination of PR and CR. Day +30 OR was defined as the achievement of PR or CR at any time within 30 d after the infusion. One-year CR was defined as the achievement of CR at any time within 1 year after the infusion. All patients who achieved CR during follow-up were in PR on day 30.

### Manufacture of CAR19/IL-15-transduced NK cells from CBU

The clinical CBUs for CAR-NK production were obtained from the MD Anderson Cord Blood Bank. CBU was collected after informed consent from mothers at several hospitals and shipped to the MD Anderson Cord Bank for processing and cryopreservation following standard operating procedures. The time from collection to cryopreservation was the time from collection of CBU at the mother’s bedside to the time the cord was cryobanked. The CAR-NK cells were manufactured in the MDACC Good Manufacturing Practice facility. Briefly, the cord unit was thawed in a water bath, and NK cells were purified by CD3, CD19 and CD14 negative selection (Miltenyi beads) and cultured in the presence of engineered K562 feeder cells expressing membrane-bound IL-21 and 4-1BB ligand plus exogenous IL-2 (200 U ml^−1^). On day 6 of culture, cells were transduced with a retroviral vector encoding the anti-CD19 CAR, IL-15 and iC9 genes, generously provided by G. Dotti (University of North Carolina)^53^. The cells were expanded for an additional 9 d and harvested for fresh infusion on day 15. For a subset of patients (*n* = 17), the products were expanded for a total of 22 d. The final CAR-NK cell transduction efficiency for the infused CAR19/IL-15 NK cell product was 72.4% (range 22.7–91.1). The median CD3-positive T cell content in the infused product was 2,000 cells per kg of body weight (range 30–16,000 cells per kg of body weight).

### Analysis of serum cytokines and CAR-NK cell monitoring

The cytokine assays were performed on serum from PB samples collected from patients at multiple time points after CAR-NK cell infusion using the Procartaplex kit from Thermo Fisher following the manufacturer’s instructions. The qPCR assays were performed on serial PB samples as previously described^13^.

### DSA measurement

Patients were screened for the presence of donor-specific HLA antibodies at the MD Anderson HLA laboratory before and at multiple time points after CAR-NK cell infusion. If the screen was positive, we determined the specificity of the antibody using a semiquantitative solid-phase fluorescent beads assay on the Luminex platform. Results were expressed as MFI with values ≥ 1,000 being considered positive.

### Cell lines, primary cells and culture conditions

Cell lines of Raji (CCL-86), MM1S (CRL-2974), SKOV3 (HTB-77), K562 (CRL-3344) and 293T (CRL-3216) were obtained from the American Type Culture Collection. Cells of Raji, MM1S and K562 were cultured in RPMI-1640 (Invitrogen) supplemented with 10% FBS (HyClone), 1% penicillin–streptomycin and 1% GlutaMAX; 293T and SKOV3 cells were cultured in DMEM (Invitrogen) supplemented with 10% FBS, 1% penicillin–streptomycin and 1% GlutaMAX. Raji cells were transduced with mCherry to facilitate their detection in in vitro assays; Raji, MM1S and SKOV3 cells were labeled with firefly luciferase (Ffluc)-GFP for in vivo tumor analysis by the IVIS Spectrum bioluminescence imaging system (Caliper). All cells were maintained in a 37 °C incubator with 5% CO_2_, and regularly tested for mycoplasma contamination using the MycoAlert Mycoplasma Detection Kit (Lonza).

### NRBC isolation from CBU

CBUs were provided by the MD Anderson CB Bank under institutional review board-approved protocols. CBMCs were isolated by a density-gradient technique (Ficoll-Histopaque; Sigma). NRBCs were isolated by positive selection using CD71 and CD235A (Glycophorin A) beads (Miltenyi Biotec) and cultured in 48-well plates at a concentration of 500,000 cells per ml in RPMI/Click’s media. Supernatants were collected for ELISA assays after 24, 48 and 72 h of culture.

### TGF-β milliplex assay

TGF-β1 and TGF-β2 measurement was performed using MILLIPLEX MAP TGFβ-3 Plex (TGFBMAG-64K-03) following the manufacturer’s instructions, on a Luminex 200 instrument. The levels of TGF-β1 and TGF-β2 in media alone were subtracted from the values obtained from NRBC conditions. Data were analyzed using Bio-Plex software.

### Arginase-1 ELISA assay

Arginase-1 quantification was performed using the BMS2216 ELISA kit from Invitrogen, following the manufacturer’s instructions. Data were acquired on a 96-well microplate reader.

### Flow cytometry

In the clinical product, CAR expression was evaluated using biotin-conjugated human CD19 CAR detection reagent (Miltenyi Biotec; 1:50 dilution) and anti-biotin antibody-APC (Miltenyi Biotec, REA746; 1:50 dilution). For negative controls, ex vivo expanded NT-NK cells from the corresponding CBUs of the CAR-NK products were used.

CAR expression in the in vitro preclinical studies was measured using a conjugated goat anti-human lgG Alexa Fluor 647 (H + L; Jackson ImmunoResearch) that recognized the IgG hinge portion of the CAR construct. We used Ghost Dye Violet 450 (Tonbo Biosciences) to determine viability, and aqua fixable viability dye (eBioscience) when fixation protocols were applied. Human Fc receptor blocking solution (Miltenyi Biotec) was applied to minimize nonspecific staining. Cell counts were measured by AccuCheck Counting Beads (Thermo Fisher). Cells were acquired on an LSRFortessa X-20 (BD Biosciences), and data were analyzed using FlowJo (version 10.8.1, BD Biosciences).

Flow cytometry antibodies for the in vivo mouse models included: Live Dead-BV510 (Invitrogen; 1:200 dilution), Human CD45-PerCP (BioLegend, HI30; 1:50 dilution), Mouse CD45-BV650 (BioLegend, 30-F11; 1:50 dilution), Human CD56-BV605 (BioLegend, 5.1H11; 1:50 dilution), Human CD16-BV605 (BioLegend, 3G8; 1:50 dilution), Human CD3-APCY7 (BioLegend, HIT3a; 1:100 dilution), Human CD19-PECY7 (BD Biosciences, SJ25C1; 1:50 dilution), Human CD20-AF700 (BD Biosciences, 2H7; 1:50 dilution), anti-biotin-APC (Miltenyi Biotec, Bio3-18E7; 1:20 dilution), CD19 CAR Detection reagent-Unconjugated (Miltenyi Biotec; 1:50 dilution), Human CD27-PECF594 (BD Biosciences, M-T271; 1:50 dilution), Human CD70-PECY7 (BioLegend, 113-16; 1:50 dilution), Human BCMA-PE (Miltenyi Biotec, REA315; 1:50 dilution), Human CD138-AF700 (BD Biosciences, MI15; 1:50 dilution), TROP2-PE (BioLegend, NY18; 1:50 dilution) and Anti-His-APC (BioLegend, J095G46; 1:50 dilution).

In the Raji mouse model, the NK cell population was identified by first gating on lymphocytes using forward and side scatters. We next gated on singlets, followed by live cells defined as Live Dead^low^. Human NK cells were identified by first gating on hCD45^+^mCD45^−^ followed by CD16^+^CD56^+^GFP^−^ cells. CAR19^+^ NK cells were identified using conjugated goat anti-human lgG; fluorescence minus one or NT-NK cells were used as controls. To identify Raji cells, we first gated on the hCD45^+^mCD45^−^ population, followed by CD16^−^CD56^−^CD19^+^GFP^+^ cells. In the MM1S mouse model, the NK cell population was identified by first gating on lymphocytes using forward and side scatters, then on singlets, followed by Live Dead^low^, then mCD45^−^CD138^−^ and finally CD16^+^CD56^+^ cells. CAR70^+^ NK cells were identified as CD16^+^CD56^+^CD27^+^, with fluorescence minus one or NT-NK cells used as controls. MM1S cells were gated from the Live Dead^low^ population and identified as hCD45^−^CD138^+^.

To evaluate trogocytosis, we measured CD19 expression on CAR-NK cells by flow cytometry in patient samples for up to 4 weeks following CAR-NK cell infusion. High trogocytosis was defined as a normalized tCD19 MFI level greater than the mean, while low trogocytosis was defined as a level equal or less than the mean at more than one time point as previously described^22^.

### Tumor rechallenge assay in IncuCyte system

NK cells were co-cultured at different E:T ratios with Raji tumor cells labeled with mCherry, and fresh tumor cells were added to the co-culture every 2–3 d. For rechallenge assays using CAR-NK cells, 100,000 mCherry-labeled Raji cells were added at each challenge. For rechallenge assays using NT-NK cells, 16,700 mCherry-labeled Raji cells were added at each challenge. The tumor cell index represents the counts of tumor cells where the intensity of mCherry fluorochrome was detected. Images of each well were captured in real time. Data were analyzed using the IncuCyte Live-Cell Imaging System that measures the number of target cells (fluorochrome labeled) in real time.

### NK PD assay

NK cells were subcultured every week, with or without K562-based feeder cells, after the initial transduction and expansion. Using the equation for PD = log_10_[(A/B)/2], where A is the number of harvested cells and B is the number of plated cells from each subculture, the weekly PD was measured, then, the sum of each PD over time was determined as the cumulative PD. Assays were terminated 3 weeks after the cell count from the subculture failed to achieve at least an equal amount of seeded cells. Data were obtained from three different CBU-derived NK populations for each condition.

### Mass cytometry (CyTOF)

Mass cytometry was performed as previously described^54^. Primary antibodies were conjugated in-house with the corresponding metal tags using MaxparX8 and MCP9 polymer antibody labeling kits per the manufacturer’s protocol (Standard BioTools). NK cells were washed with cell staining buffer (0.5% BSA/PBS). Cells were then incubated with 2.5 μM cisplatin (Pt198, Standard BioTools) for 3 min for viability assessment, followed by washing twice with cell staining buffer. Cells were then stained with freshly prepared antibody mix against cell surface markers for 30 min on a shaker at room temperature, then washed twice and fixed with freshly prepared 1.6% paraformaldehyde (EMD Biosciences)/PBS for 10 min at room temperature. The cells were then rinsed twice with cell staining buffer and incubated overnight in −80 °C with 80% methanol. The following day, the cells were stained with intracellular marker-specific antibodies for 45–60 min in the presence of 0.2% saponin. After an additional washing step, the cells were stored overnight in 1,000 μl Maxpar fix and perm buffer (Standard BioTools, 201067) with 125 nM of Iridium nucleic acid intercalator (Standard BioTools) in 4 °C. The cells were then washed and resuspended in MilliQ dH_2_O supplemented with EQTM 4-element calibration beads, and subsequently acquired at 300 events per second on a Helios instrument (Standard BioTools). The CyTOF antibodies used with the corresponding metal tag isotopes are: CD45 (Standard BioTools, HI30, ^89^Y; 1:200 dilution), CCR6 (Miltenyi Biotec, REA190, ^141^Pr; 1:125 dilution), EOMES (Invitrogen, WD1928, ^142^Nd; 1:200 dilution), KIR2DL4 (Miltenyi Biotec, REA768, ^143^Nd; 1:250 dilution), KIR3DL1 (BD Pharmingen, DX9, ^144^Nd; 1:300 dilution), CD70 (BioLegend, 113−16, ^145^Nd; 1:500 dilution), KIR2DL5 (Miltenyi Biotec, REA955, ^146^Nd; 1:125 dilution), NKG2C (Miltenyi Biotec, REA205, ^147^Sm; 1:125 dilution), TRAIL (Miltenyi Biotec, REA1113, ^148^Nd; 1:125 dilution), CD25 (Standard BioTools, 2A3, ^149^Sm; 1:125 dilution), CD69 (Miltenyi Biotec, REA824, ^150^Nd; 1:5,000 dilution), 2B4 (Miltenyi Biotec, REA112, ^151^Eu; 1:5,000 dilution), granzyme B (GrB; Miltenyi Biotec, REA226, ^152^Sm; 1:5,000 dilution), TIM3 (Miltenyi Biotec, REA635, ^153^Eu; 1:125 dilution), CX3CR1 (Miltenyi Biotec, REA385, ^154^Sm; 1:125 dilution), KIR2DL3 (Miltenyi Biotec, REA147, ^155^Gd; 1:200 dilution), CXCR3 (Standard BioTools, G025H7, ^156^Gd; 1:200 dilution), OX40 (Miltenyi Biotec, REA621, ^158^Gd; 1:125 dilution), PFN (Miltenyi Biotec, REA1061, ^159^Tb; 1:5,000 dilution), T-bet (Standard BioTools, 4B10, ^160^Gd; 1:250 dilution), TIGIT (Miltenyi Biotec, REA1004, ^161^Dy; 1:125 dilution), Ki67 (Standard BioTools, B56, ^162^Dy; 1:250 dilution), KIR2DL1 (Miltenyi Biotec, REA284, ^163^Dy; 1:250 dilution), KIR2DS1 (R&D Systems, 1127B, ^164^Dy; 1:250 dilution), PD1 (Miltenyi Biotec, PD1.3.1.3, ^165^Ho; 1:125 dilution), NKG2D (Miltenyi Biotec, REA797, ^166^Er; 1:300 dilution), CD38 (Miltenyi Biotec, REA572, ^167^Er; 1:500 dilution), CD73 (Standard BioTools, AD2, ^168^Er; 1:100 dilution), CD39 (Miltenyi Biotec, MZ18-23C8, ^169^Tm; 1:200 dilution), CD161 (Miltenyi Biotec, REA631, ^170^Er; 1:500 dilution), DNAM (Miltenyi Biotec, REA1040, 171Yb; 1:250 dilution), KLRG1 (Miltenyi Biotec, REA261, ^172^Yb; 1:125 dilution), CXCR4 (Standard BioTools, 12G5, ^173^Yb; 1:200 dilution), KIR2DS4 (Miltenyi Biotec, REA860, ^174^Yb; 1:250 dilution), LAG3 (Miltenyi Biotec, REA351, ^175^Lu; 1:125 dilution), ICOS (Miltenyi Biotec, REA192, ^176^Yb; 1:200 dilution), CD16 (Standard BioTools, 3G8, ^209^Bi; 1:200 dilution), CD57 (Miltenyi Biotec, REA769, ^115^In; 1:500 dilution), CD3 (Miltenyi Biotec, REA613, ^194^Pt; 1:250 dilution), NKG2A (Miltenyi Biotec, REA110, ^195^Pt; 1:500 dilution), HLA-DR (Miltenyi Biotec, REA805, ^196^Pt; 1:250 dilution), LD (Standard BioTools, Cisplatin, ^198^Pt, 2.5 μM), CD56 (Miltenyi Biotec, REA196, ^106^Cd; 1:200 dilution), CAR (Miltenyi Biotec, REA1298, ^110^Cd; 1:50 dilution), CD2 (Miltenyi Biotec, REA972, ^111^Cd; 1:300 dilution), CD8 (Miltenyi Biotec, REA734, 112 Cd; 1:250 dilution), NKP30 (Miltenyi Biotec, AF29-4D12, ^113^Cd; 1:200 dilution), NKP46 (Miltenyi Biotec, REA808, ^114^Cd; 1:125 dilution), NKP44 (Miltenyi Biotec, REA1163, ^116^Cd; 1:125 dilution), CD36 (Miltenyi Biotec, REA760, ^142Nd^; 1:300 dilution), CD127 (Standard BioTools, A019D5, ^143^Nd; 1:200 dilution), CD11b (Standard BioTools, ICRF44, ^144^Nd; 1:250 dilution), CD62L (Miltenyi Biotec, REA615, ^145^Nd; 1:200 dilution), CD64 (Miltenyi Biotec, REA978, ^148^Nd; 1:250 dilution), CD86 (Miltenyi Biotec, REA968, ^150^Nd; 1:125 dilution), CD123 (Miltenyi Biotec, REA918, ^151^Eu; 1:250 dilution), TCRgd (Miltenyi Biotec, REA591, ^152^Sm; 1:250 dilution), CD27 (Miltenyi Biotec, REA499, ^155^Gd; 1:250 dilution), CCR4 (Miltenyi Biotec, REA279, ^158^Gd; 1:125 dilution), CD11c (Standard BioTools, Bu15, ^159^Tb; 1:250 dilution), CD80 (Standard BioTools, 2D10.4, ^161^Dy; 1:125 dilution), CD66B (Standard BioTools, 80H3, ^162^Dy; 1:250 dilution), TCR Va7.2 (Miltenyi Biotec, REA179, ^163^Dy; 1:200 dilution), CD45RO (Miltenyi Biotec, REA611, ^164^Dy; 1:200 dilution), CD163 (Standard BioTools, GHI/61, ^165^Ho; 1:200 dilution), CCR7 (Miltenyi Biotec, REA546, ^167^Er; 1:200 dilution), CD45RA (Miltenyi Biotec, REA562, ^169^Tm; 1:200 dilution), CXCR5 (Miltenyi Biotec, REA103, 171Yb; 1:300 dilution), iNKT (BioLegend, 6B11, ^173^Yb; 1:200 dilution), CD95 (Standard BioTools, DX2, ^175^Lu; 1:250 dilution), CD19 (Miltenyi Biotec, REA675, ^110^Cd; 1:200 dilution), CD4 (Miltenyi Biotec, REA623, ^111^Cd; 1:300 dilution), CD15 (BD Pharmingen, HI98, ^113^Cd; 1:500 dilution), CD14 (Miltenyi Biotec, REA599, ^114^Cd; 1:200 dilution), CD20 (Miltenyi Biotec, REA780, ^116^Cd; 1:200 dilution), GFP (BioLegend, FM264G, ^144^Nd; 1:250 dilution), CD81 (Miltenyi Biotec, REA513, ^145^Nd; 1:250 dilution), PANKIR (R&D, 180704, ^153^Eu; 1:300 dilution), mCD45 (BioLegend, 30-F11, ^154^Sm; 1:125 dilution), PFN (Standard BioTools, B-D48, ^196^Pt; 1:200 dilution) and GrB (Standard BioTools, GB11, ^198^Pt; 1:200 dilution).

### Mass cytometry data analysis

Mass cytometry data were analyzed using Cytobank. The NK cell population was identified using the following gating strategy: gating singlets followed by Pt198 (cisplatin)^low^ followed by hCD45^+^CD56^+^CD3^−^. The gating strategy was applied to all files. CAR^+^ NK cells were determined compared to either isotype controls or NT-NK cell controls. NK cells from each donor were downsampled in FlowJo using the Downsample plugin. Normalized data were pooled according to Opt-Cs-versus-Sub-Cs classification and analyzed together in Cytobank. SPADE analysis was performed for clustering and visualization of high-dimensional single-cell data. Cells with phenotypical similarity were hierarchically clustered together in subclusters (nodes) that form clusters (branches) to indicate the diverse phenotypic landscape of the data. The expression of each marker in the subclusters was transformed and normalized locally and plotted as a heat map using Morpheus matrix visualization and analysis software (Broad Institute).

### Seahorse metabolic assays

The ECAR (surrogate for glycolysis) and OCR (surrogate for mitochondrial function) were measured using the Agilent Seahorse XF Pro Analyzer (Agilent) following the manufacturer’s protocol. ECAR was measured by Seahorse Glycolysis Stress Test using 2 g l^−1^ d-glucose, 2.5 μM oligomycin and 50 mM 2-deoxyglucose mixed with Hoechst 33342 (Invitrogen) dye. OCR was measured by Seahorse Mito Stress Test using 2.5 μM oligomycin, 0.5 μM FCCP and 0.5 μM rotenone/antimycin A mixed with Hoechst 33342 (Invitrogen) dye. Each NK cell condition was assayed in technical triplicates. Following the assays, live-cell imaging and viable cell counting were performed in a Cytation 1 machine. Normalized OCR or ECAR data per 250,000 live cells were shown. The basal respiration was calculated as follows: last rate measurement before first injection − non-mitochondrial respiration rate, which represents the minimum rate measurement after rotenone/antimycin A. The maximal respiration was calculated as follows: maximum rate measurement after FCCP injection − non-mitochondrial respiration. The baseline glycolysis was presented as the non-glycolytic acidification, which consists of the last rate measurement before glucose injection. The glycolytic capacity was calculated as follows: maximum rate measurement after oligomycin injection − last rate measurement before glucose injection.

### IsoPlexis assays

The single-cell secretome analysis was performed using the IsoCode chip from IsoPlexis using the human NK cytokine panel. The assay was performed using the manufacturer’s kit and following the manufacturer’s instructions (IsoPlexis). In brief, NT-NK cells were stimulated using purified anti-human CD16 (BD Pharmingen, 555404; 1 μg ml^−1^) and CAR-NK cells were stimulated using human CD19 antigen (ACRO, CD9-H5259; 10 μg ml^−1^) for 4 h at 37 °C. NK cells were washed and labeled with a fluorescent dye (IsoPlexis stain cell membrane 405), and 30,000 cells were loaded onto the IsoCode chips. The IsoLight device was used to scan the chips, and IsoPlexis’s proprietary IsoSpeak software was used to analyze the data. The PSI, as computed by the software, was used for data representation. Stimulatory cytokines comprised GM-CSF, IL-12, IL-15, IL-2, IL-21, IL-5, IL-7, IL-8 and IL-9. Effector cytokines comprised GZMB, IFN-γ, MIP-1α, perforin, TNF-α and TNF-β. Chemokines comprised CCL-11, IP-10, MIP-1β and RANTES^55^.

### Bulk RNA-seq processing and differential expression

Cord units (Supplementary Table 2) were thawed, NK cells were purified using NK negative selection beads (Miltenyi beads) and sequencing was performed in the MDACC Genomics Core and at Avera Institute for Human Genetics. Sequencing at the MDACC Genomics Core was done as follows: Stranded mRNA libraries were prepared using the KAPA Stranded mRNA-Seq Kit (Roche). Briefly, PolyA RNA was captured from 250 ng of total RNA using magnetic Oligo-dT beads. After bead elution and cleanup, the resultant PolyA RNA was fragmented using heat and magnesium. First-strand synthesis was performed using random priming followed by second-strand synthesis with the incorporation of deoxyuridine triphosphate (dUTP) into the second strand. The ends of the resulting double-stranded cDNA fragments were repaired, 5′-phosphorylated and 3′-A tailed and Illumina-specific indexed adaptors were ligated. The products were purified and enriched for a full-length library with 12 cycles of PCR. The strand marked with dUTP is not amplified, resulting in a strand-specific library. The libraries were quantified using the Qubit dsDNA HS Assay Kit (Thermo Fisher) and assessed for size distribution using the 4200 Agilent TapeStation (Agilent Technologies). Equimolar quantities of the indexed libraries were then multiplexed, with 12 libraries per pool. The library pool was quantified by qPCR, then sequenced on the Illumina NextSeq 500 high-output 150 flow cell using the 75-nucleotide paired-end format. Sequencing at Avera Institute for Human Genetics was done as follows: A total of 21 isolated total RNAs were assessed for concentration and integrity on an RNA 6000 Nano chip ran on a 2100 BioAnalyzer (Agilent) where the average RNA integrity score was 8.4 and the average concentration was 16.0 ng µl^−1^. A sample input amount of 100 ng of total RNA was utilized for each sample for library preparation using the Illumina Stranded mRNA Library Prep Kit (Illumina). Briefly, polyA mRNA was captured utilizing oligo (dT) magnetic beads, fragmented appropriately and primed for cDNA synthesis with random hexamers. Blunt-ended cDNA was generated after first-strand and second-strand synthesis where the addition of dUTP is incorporated to achieve strand specificity. Adenylation of the 3′ blunt ends was followed by pre-index anchor ligation before the enrichment of the cDNA fragment with indexed primer sequences. Final library quality control was carried out by evaluating the fragment size on a DNA1000 chip ran on a 2100 BioAnalyzer (Agilent). The concentration of each library was determined by qPCR using the KAPA Library Quantification Kit for Next Generation Sequencing (KAPA Biosystems) before sequencing. The average concentration of the final library was determined to be 82.8 nM. Libraries were normalized to 2 nmol l^−1^ in RSB/Tween 20 and then pooled evenly. The library pool along with a 0.5% PhiX control was loaded onto Illumina’s NextSeq 2000 Sequencing System where denaturation and cluster generation were performed according to the manufacturer’s specifications (Illumina). Sequencing by synthesis was performed on a NextSeq 2000 in a 2 × 100 fashion utilizing v3 chemistry with a P1 flow cell, which resulted in an average of 24 million paired-end reads per sample. Sequence read data were processed and converted to FASTQ format for downstream analysis by Illumina BaseSpace software, BCL Convert 3.8.4.

Fastq file quality control was performed with FASTQC (https://www.bioinformatics.babraham.ac.uk/projects/fastqc/) using the R package fastqcr (https://cran.r-project.org/web/packages/fastqcr/index.html). Gene expression was quantified with RSEM^56^ (v1.3.3; rsem-calculate-expression --strandedness reverse --no-bam-output --paired-end --bowtie2) using the bowtie2 (v2.4.2)^57^ as the aligner and hg19 transcriptome as the reference. TPM values from RSEM were log_2_ transformed (log_2_(TPM + 1)) and the top 5,000 variably expressed genes were used to perform PCA and visualize clustering of the samples.

Differential expression analysis comparing NK cells from Opt-Cs to Sub-Cs was performed using DESeq2 (ref. ^58^) with the counts imported from the output of RSEM using tximport^59^. The differential expression model controlled for batch if samples in a comparison came from multiple sequencing batches. Differentially expressed genes were identified at adjusted *P* value < 0.1 and absolute log_2_ fold change > 1.5.

Differential pathway analysis was performed using GSEA, implemented in the Bioconductor gage^60^, using ordered gene lists. The gene lists were ordered by ‘stat’ column of Deseq2’s output. Differentially activated pathways were identified at *q* value < 0.1, with positive mean statistic indicating upregulation in Opt-Cs and negative values indicating upregulation in Sub-Cs. Enrichment plots were generated using the GSEA tool (https://www.gsea-msigdb.org/gsea/index.jsp). The hallmark^61^ pathway definitions were used for GSEA.

### NK functional score

Activity of NK function signature (*GZMA*, *PRF1*, *GZMB* and *CD247*) was estimated in each sample using ssGSEA^62^ implemented in the R package GSVA^63^. The difference between Opt-Cs and Sub-Cs was computed using a two-tailed Student’s *t*-test.

### Bulk RNA-seq regulon analysis

To identify key TFs and measure the activity of regulons in bulk RNA-seq data, we used the pySCENIC workflow described previously^64^. We applied the default pySCENIC parameters on a high-performance computing system to infer regulatory interactions between predefined lists of TFs and candidate target genes. pySCENIC utilizes the gradient boosting machine regression GRNBoost2 algorithm and Arboreto library^65^ to calculate coexpression patterns from transcriptomics data. This results in an adjacencies matrix connecting each TF with its target gene(s) along with an importance score, which separates high-confidence interactions from the weak ones. To generate candidate modules, we selected TF–target gene interactions and assembled them into modules consisting of target genes that would be regulated by a given TF, also referred to as regulons. We further refined these modules by separating the direct targets of a given regulator from the indirect ones. This was achieved by identifying target genes that have the DNA motif specific to a certain TF in their promoter region. To do this, we used *cis*-regulatory module scoring with RcisTarget, which looks for modules with cisTarget motif enrichment using precomputed whole-genome rankings of all motifs linked to known TFs in the pySCENIC database. We then calculated the AUC scores to measure the biological activity of each regulon at the sample level. We identified differentially active regulons in NK cells between Opt-C and Sub-C samples at the prestimulation time point using a *t*-test, which was corrected for multiple-hypothesis testing using Bonferroni correction. We used an adjusted *P* value < 0.01 to display statistically significant hits on the scaled regulon activity scores and compared them between different conditions.

### Bulk ATAC-seq analysis

ATAC-seq library preparation was performed at the MDACC Epigenomics Profiling Core following the protocol previously described^66,67^ with minor modifications. Briefly, nuclei isolated from NK cells derived from nine donors (Supplementary Table 2) were tagmented using Tagment DNA enzyme (Illumina) and the resulting libraries were purified using SPRISelect beads (Beckman Coulter). Libraries were sequenced (2 × 100 bp) on an Illumina NovaSeq 6000 to obtain at least 50 million high-quality mapping reads per sample.

For each bulk ATAC-seq sample, the pair-end reads from fastq files were aligned to the human genome (GRCh38) using bwa mem mode with duplicated reads removed^68^. The 5′ ends of ATAC-seq reads were shifted to the actual cut site of the transposase using the alignmentSieve module implemented in DeepTools^69^. The peaks were called with MACS2 (ref. ^70^) using the pair-end read information. The samples had a comparable total number of reads: mean = 1.05 × 10^6^, s.d. = 0.11 × 10^6^ in the Sub-C samples; and mean = 1.19 × 10^6^, s.d. = 0.07 × 10^6^ in the Opt-C samples. The minimum false discovery rate (*q* value) cutoff for peak detection was set as 0.05. The MACS2 outputs from multiple samples were loaded using DiffBind^71^. The peak sets from multiple samples were identified as the overlapping ones among samples using bUseSummarizeOverlaps function in DiffBind. We then calculated the TF activity level using the function RunChromVAR in Signac^72^ and gene-level accessibility level using the geneActivity function in Seurat. We identified TFs of significantly different activity levels using a two-tailed Wilcoxon rank-sum test. The difference between Opt-Cs and Sub-Cs on peak, gene accessibility and motif-based TF activity levels was identified using the function FindMarkers in Signac^72^. For Opt-Cs and Sub-Cs upregulated peaks, the motif enrichments were performed using HOMER^73^ with statistical significance estimated using a one-tailed binomial distribution test. The peak track profiles of candidate genes were visualized using the online Integrative Genomics Viewer tool (for replicates from the same group, the peak track profiles were aggregated).

### Viral constructs and retrovirus production

The CAR targeting CD70 construct (iC9.CD27(ECD).CD28.zeta.2 A.IL-15) referred to as CAR70/IL-15 incorporates the CD27 extracellular domain (which naturally binds to CD70), linked to the CD28 costimulatory domain and the CD3ζ signaling domain. Additionally, it includes iC9 as a safety switch and the IL-15 transgene.

The CAR targeting TROP2 construct (iC9.TROP2scFv (clone hRS7).CD28.zeta.2 A.IL-15) referred to as CAR-TROP2/IL-15 consists of an scFv targeting TROP2 (derived from the human RS7 sequence of the TROP2-targeting antibody–drug conjugate sacituzumab govitecan), coupled with the CD28 costimulatory domain and the CD3ζ signaling domain. Similarly, the construct includes iC9 as a safety switch and IL-15.

The CAR70/IL-15 and CAR-TROP2/IL-15 constructs were cloned into the SFG retroviral backbone to generate viral vectors. Transient retroviral supernatants were produced from transfected 293T cells as previously described^74^.

### Xenogeneic tumor-grafted mouse models

NOD/SCID IL-2Rγ^null^ (NSG) mice engrafted with different tumor cell lines were used to examine the antitumor activity of the different CAR-NK cell products. Tumor models included Raji lymphoma, MM1S multiple myeloma and SKOV3 ovarian cancer. All experiments were performed in accordance with the American Veterinary Medical Association and National Institutes of Health (NIH) recommendations under protocols approved by the MDACC Institutional Animal Care and Use Committee (protocol no. 00000889-RN02). Mice were maintained under specific-pathogen-free conditions, with a 12-h night–day cycle of light, and at a stable ambient temperature with 40–70% relative humidity. We utilized an aggressive NK-resistant Raji NSG (The Jackson Laboratory) xenograft model. Ten-week-old male mice were irradiated on day −1 and engrafted with Ffluc-Raji cells (0.2 × 10^5^). CAR19/IL-15 CBU-NK cells from Opt-Cs or Sub-Cs were injected via tail vein when indicated. Weekly bioluminescence imaging (Xenogen IVIS-200 Imaging System) was performed to monitor tumor growth. Flow cytometry was used to measure NK cell trafficking, persistence and expansion. We utilized a second mouse model of MM1S to validate the results. Ten-week-old female mice were irradiated on day −4 and engrafted with Ffluc-MM1S (5 × 10^5^) on day −3. CAR70/IL-15-transduced CBU-NK cells from Opt-Cs or Sub-Cs were injected via tail vein when indicated. Mice were subjected to weekly bioluminescence imaging. Trafficking, persistence and expansion of NK cells were measured by flow cytometry. For the ovarian cancer model SKOV3, nine-week-old female mice were injected with Ffluc-SKOV3 (5 × 10^5^) on day −7 intraperitoneally, and mice were irradiated on day −1. CAR-TROP2/IL-15-transduced CBU-NK cells from Opt-Cs or Sub-Cs were injected intraperitoneally on day 0. Mice were subjected to weekly bioluminescence imaging (Xenogen IVIS-200 Imaging System) and data were analyzed using Living Image v4.4.

### Statistical methods

The statistical rationale for the sample size of patients enrolled on the trial was not based on a power computation. Rather, the reliability of Bayesian posterior estimators of probability (efficacy) and probability (toxicity) was quantified by assuming a non-informative prior for each probability and computing a posterior 95CrI^75,76^. CR and OR were reported as cumulative response rates. Probabilities of 1-year OS and PFS were calculated using the Kaplan–Meier method. For the PFS analysis, death for any reason, progression of the disease or loss of a previously achieved response were considered as the events of interest. Survival times were censored at last patient follow-up. The influence of variables on the proportion of day +30 OR or 1-year CR was examined with the Fisher’s exact test. OS and PFS were compared using the log-rank test. Bayesian methods were used for multiple regression^76,77^. For regression of binary outcomes on patient covariates, a logistic model was assumed. For regression of each outcome on patient covariates, independent non-informative normal (0, 10) priors were assumed for all covariate parameters. In each regression model, the effect of each covariate with coefficient b on the outcome was quantified by the posterior PBE = Pr(b > 0 | data). A PBE near 0 implies a very harmful effect of the covariate on the outcome, a PBE near 1 implies a very beneficial effect of the covariate, and PBE = 0.50 corresponds to no effect. For PFS or OS, PBE = Pr(HR < 1 | data) = the probability of a lower risk of the failure event for the covariate. We used the Wilcoxon rank-sum or the Kruskal–Wallis test to study the association between the copy number by qPCR of CAR-NK cells and other variables. The Student’s *t*-test, one-way ANOVA and two-way ANOVA were used for the in vitro and in vivo mouse studies as indicated. For the comparison of survival curves in the mouse experiments, the Kaplan–Meier method and log-rank test were used. Sample sizes were estimated based on preliminary experiments. Power calculations predicted at least 80% power to detect a relative HR of 4.3–6 between two groups at the significance level of 0.05. The power was calculated based on the proportional-hazards regression model under the assumption that HR between any two mice is constant over the entire duration of the study^78^. All reported *P* values are two-tailed and *P* values of less than 0.05 were considered significant. The analyses were performed using SPSS version 26.0, R version 4.2.1, JAGS version 4.3.1 and GraphPad Prism version 7.0.

### Reporting summary

Further information on research design is available in the Nature Portfolio Reporting Summary linked to this article.

## Online content

Any methods, additional references, Nature Portfolio reporting summaries, source data, extended data, supplementary information, acknowledgements, peer review information; details of author contributions and competing interests; and statements of data and code availability are available at 10.1038/s41591-023-02785-8.

### Supplementary information

Supplementary Information Supplementary Figs. 1–4 and Supplementary Tables 1 and 2.

Reporting Summary

Supplementary Data Statistical source data for Supplementary Figs. 1–3.

### Source data

Source Data Fig. 1 Statistical source data.

Source Data Fig. 2 Statistical source data.

Source Data Fig. 3 Statistical source data.

Source Data Extended Data Fig. 2 Statistical source data.

Source Data Extended Data Fig. 3 Statistical source data.

Source Data Extended Data Fig. 4 Statistical source data.

Source Data Extended Data Fig. 6 Statistical source data.

Source Data Extended Data Fig. 7 Statistical source data.

Source Data Extended Data Fig. 8 Statistical source data.

Source Data Extended Data Fig. 9 Statistical source data.

## Data Availability

ATAC-seq and RNA-seq data are available through the Gene Expression Omnibus (https://www.ncbi.nlm.nih.gov/geo/) under accession number GSE233149. The pySCENIC database is available through the pySCENIC cisTarget database: https://resources.aertslab.org/cistarget/databases/homo_sapiens/hg38/refseq_r80/mc9nr/gene_based/. The data reported in this article are commercially sensitive and not publicly available. To the extent allowed, the authors will provide access to deidentified participant-level data underlying the data presented in this article to researchers who provide a methodologically sound proposal for academic purposes to interpret, verify and extend research in the article that does not violate privacy, data encumbrance, intellectual property or other legal, regulatory or contractual confidentiality obligations, beginning 12 months after article publication. Data provided will be subject to a data use agreement. Researchers should contact the corresponding author when applying for data access. Response to external data requests will be within a reasonable timeframe of a few weeks to months depending on the nature of the request. Use of data will be restricted to the agreed purpose. Source data are provided with this paper.

## Code availability

No custom code was used.

## References

[1] Abramson JS (2020). Lisocabtagene maraleucel for patients with relapsed or refractory large B-cell lymphomas (TRANSCEND NHL 001): a multicentre seamless design study. Lancet.

[2] Neelapu SS (2017). Axicabtagene ciloleucel CAR T-cell therapy in refractory large B-cell lymphoma. N. Engl. J. Med..

[3] Schuster SJ (2019). Tisagenlecleucel in adult relapsed or refractory diffuse large B-cell lymphoma. N. Engl. J. Med..

[4] Wang M (2020). KTE-X19 CAR T-cell therapy in relapsed or refractory mantle-cell lymphoma. N. Engl. J. Med..

[5] Brudno JN, Kochenderfer JN (2019). Recent advances in CAR T-cell toxicity: mechanisms, manifestations and management. Blood Rev..

[6] Laskowski TJ, Biederstädt A, Rezvani K (2022). Natural killer cells in antitumour adoptive cell immunotherapy. Nat. Rev. Cancer.

[7] Cerwenka A, Lanier LL (2016). Natural killer cell memory in infection, inflammation and cancer. Nat. Rev. Immunol..

[8] Huntington ND, Cursons J, Rautela J (2020). The cancer–natural killer cell immunity cycle. Nat. Rev. Cancer.

[9] Vivier E (2011). Innate or adaptive immunity? The example of natural killer cells. Science.

[10] Daher M, Rezvani K (2021). Outlook for New CAR-based therapies with a focus on CAR NK cells: what lies beyond CAR-engineered T cells in the race against cancer. Cancer Discov..

[11] Rafei H, Daher M, Rezvani K (2021). Chimeric antigen receptor (CAR) natural killer (NK)-cell therapy: leveraging the power of innate immunity. Br. J. Haematol..

[12] Liu E (2018). Cord blood NK cells engineered to express IL-15 and a CD19-targeted CAR show long-term persistence and potent antitumor activity. Leukemia.

[13] Liu E (2020). Use of CAR-transduced natural killer cells in CD19-positive lymphoid tumors. N. Engl. J. Med..

[14] Fraietta JA (2018). Determinants of response and resistance to CD19 chimeric antigen receptor (CAR) T cell therapy of chronic lymphocytic leukemia. Nat. Med..

[15] Burnham RE (2020). Characterization of donor variability for γδ T cell ex vivo expansion and development of an allogeneic γδ T cell immunotherapy. Front. Med..

[16] Jonus HC (2022). Dissecting the cellular components of ex vivo γδ T cell expansions to optimize selection of potent cell therapy donors for neuroblastoma immunotherapy trials. Oncoimmunology.

[17] Longo DM (2012). Inter-donor variation in cell subset specific immune signaling responses in healthy individuals. Am. J. Clin. Exp. Immunol..

[18] Belderbos ME (2020). Donor-to-donor heterogeneity in the clonal dynamics of transplanted human cord blood stem cells in murine xenografts. Biol. Blood Marrow Transpl..

[19] Morgan CJ (2019). Landmark analysis: a primer. J. Nucl. Cardiol..

[20] Bansal A, Heagerty PJ (2019). A comparison of landmark methods and time-dependent ROC methods to evaluate the time-varying performance of prognostic markers for survival outcomes. Diagn. Progn. Res..

[21] Hamieh M (2019). CAR T cell trogocytosis and cooperative killing regulate tumour antigen escape. Nature.

[22] Li Y (2022). KIR-based inhibitory CARs overcome CAR-NK cell trogocytosis-mediated fratricide and tumor escape. Nat. Med..

[23] Sotillo E (2015). Convergence of acquired mutations and alternative splicing of CD19 enables resistance to CART-19 immunotherapy. Cancer Discov..

[24] Majzner RG, Mackall CL (2018). Tumor antigen escape from CAR T-cell therapy. Cancer Discov..

[25] Goel M, Dwivedi R, Gohiya P, Hegde D (2013). Nucleated red blood cell in cord blood as a marker of perinatal asphyxia. J. Clin. Neonatol..

[26] Colaco, S. M., Ahmed, M., Kshirsagar, V. Y. & Bajpai, R. Study of nucleated red blood cell counts in asphyxiated newborns and the fetal outcome. *Int. J. Clin. Pediatr.* **3**, 79–85 (2014).

[27] Sarkar S (2013). Hypoxia induced impairment of NK cell cytotoxicity against multiple myeloma can be overcome by IL-2 activation of the NK cells. PLoS ONE.

[28] Tang F (2023). A pan-cancer single-cell panorama of human natural killer cells. Cell.

[29] Shahbaz S (2018). CD71^+^VISTA^+^ erythroid cells promote the development and function of regulatory T cells through TGF-β. PLoS Biol..

[30] Kanemasa H (2021). The immunoregulatory function of peripheral blood CD71^+^ erythroid cells in systemic-onset juvenile idiopathic arthritis. Sci. Rep..

[31] Yang J (2018). Red blood cells in Type 2 diabetes impair cardiac post-ischemic recovery through an arginase-dependent modulation of nitric oxide synthase and reactive oxygen species. JACC Basic Transl. Sci..

[32] Paul S, Lal G (2017). The molecular mechanism of natural killer cells function and its importance in cancer immunotherapy. Front. Immunol..

[33] Choi C, Finlay DK (2021). Optimising NK cell metabolism to increase the efficacy of cancer immunotherapy. Stem Cell Res. Ther..

[34] Chu Y (2023). Pan-cancer T cell atlas links a cellular stress response state to immunotherapy resistance. Nat. Med..

[35] Selli ME (2023). Costimulatory domains direct distinct fates of CAR-driven T cell dysfunction. Blood.

[36] Belk JA (2022). Genome-wide CRISPR screens of T cell exhaustion identify chromatin remodeling factors that limit T cell persistence. Cancer Cell.

[37] Maurer MJ (2018). Diagnosis-to-treatment interval is an important clinical factor in newly diagnosed diffuse large B-cell lymphoma and has implication for bias in clinical trials. J. Clin. Oncol..

[38] Cappell KM, Kochenderfer JN (2023). Long-term outcomes following CAR T cell therapy: what we know so far. Nat. Rev. Clin. Oncol..

[39] Chong EA, Ruella M, Schuster SJ (2021). Five-year outcomes for refractory B-cell lymphomas with CAR T-cell therapy. N. Engl. J. Med..

[40] Jacobson CA (2022). Axicabtagene ciloleucel in relapsed or refractory indolent non-Hodgkin lymphoma (ZUMA-5): a single-arm, multicentre, phase 2 trial. Lancet Oncol..

[41] Frey NV (2020). Long-term outcomes from a randomized dose optimization study of chimeric antigen receptor modified T cells in relapsed chronic lymphocytic leukemia. J. Clin. Oncol..

[42] Siddiqi T (2022). Phase 1 TRANSCEND CLL 004 study of lisocabtagene maraleucel in patients with relapsed/refractory CLL or SLL. Blood.

[43] Locke FL (2019). Long-term safety and activity of axicabtagene ciloleucel in refractory large B-cell lymphoma (ZUMA-1): a single-arm, multicentre, phase 1–2 trial. Lancet Oncol..

[44] Deng Q (2020). Characteristics of anti-CD19 CAR T cell infusion products associated with efficacy and toxicity in patients with large B cell lymphomas. Nat. Med..

[45] Locke FL (2020). Tumor burden, inflammation, and product attributes determine outcomes of axicabtagene ciloleucel in large B-cell lymphoma. Blood Adv..

[46] Rossi J (2018). Preinfusion polyfunctional anti-CD19 chimeric antigen receptor T cells are associated with clinical outcomes in NHL. Blood.

[47] Imai K, Matsuyama S, Miyake S, Suga K, Nakachi K (2000). Natural cytotoxic activity of peripheral-blood lymphocytes and cancer incidence: an 11-year follow-up study of a general population. Lancet.

[48] Bozorgmehr N (2023). CD71^+^ erythroid cells suppress T-cell effector functions and predict immunotherapy outcomes in patients with virus-associated solid tumors. J. Immunother. Cancer.

[49] Shanware NP (2010). Conserved and distinct modes of CREB/ATF transcription factor regulation by PP2A/B56γ and genotoxic stress. PLoS ONE.

[50] Thall PF, Simon RM, Estey EH (1995). Bayesian sequential monitoring designs for single-arm clinical trials with multiple outcomes. Stat. Med..

[51] Cheson BD (2014). Recommendations for initial evaluation, staging, and response assessment of Hodgkin and non-Hodgkin lymphoma: the Lugano classification. J. Clin. Oncol..

[52] Hallek M (2018). iwCLL guidelines for diagnosis, indications for treatment, response assessment, and supportive management of CLL. Blood.

[53] Hoyos V (2010). Engineering CD19-specific T lymphocytes with interleukin-15 and a suicide gene to enhance their anti-lymphoma/leukemia effects and safety. Leukemia.

[54] Daher M (2021). Targeting a cytokine checkpoint enhances the fitness of armored cord blood CAR-NK cells. Blood.

[55] Xue Q (2017). Single-cell multiplexed cytokine profiling of CD19 CAR-T cells reveals a diverse landscape of polyfunctional antigen-specific response. J. Immunother. Cancer.

[56] Li B, Dewey CN (2011). RSEM: accurate transcript quantification from RNA-seq data with or without a reference genome. BMC Bioinformatics.

[57] Langmead B, Salzberg SL (2012). Fast gapped-read alignment with Bowtie 2. Nat. Methods.

[58] Love MI, Huber W, Anders S (2014). Moderated estimation of fold change and dispersion for RNA-seq data with DESeq2. Genome Biol..

[59] Soneson C, Love MI, Robinson MD (2015). Differential analyses for RNA-seq: transcript-level estimates improve gene-level inferences. F1000Res.

[60] Luo W, Friedman MS, Shedden K, Hankenson KD, Woolf PJ (2009). GAGE: generally applicable gene-set enrichment for pathway analysis. BMC Bioinformatics.

[61] Liberzon A (2015). The Molecular Signatures Database (MSigDB) hallmark gene set collection. Cell Syst..

[62] Barbie DA (2009). Systematic RNA interference reveals that oncogenic KRAS-driven cancers require TBK1. Nature.

[63] Hänzelmann S, Castelo R, Guinney J (2013). GSVA: gene set variation analysis for microarray and RNA-seq data. BMC Bioinformatics.

[64] Van de Sande B (2020). A scalable SCENIC workflow for single-cell gene regulatory network analysis. Nat. Protoc..

[65] Moerman T (2018). GRNBoost2 and Arboreto: efficient and scalable inference of gene regulatory networks. Bioinformatics.

[66] Corces MR (2017). An improved ATAC-seq protocol reduces background and enables interrogation of frozen tissues. Nat. Methods.

[67] Thiyagarajan T (2023). Inhibiting androgen receptor splice variants with cysteine-selective irreversible covalent inhibitors to treat prostate cancer. Proc. Natl Acad. Sci. USA.

[68] Li, H. Aligning sequence reads, clone sequences and assembly contigs with BWA-MEM. Preprint at *arXiv*10.48550/arXiv.1303.3997 (2013).

[69] Ramírez F (2016). deepTools2: a next generation web server for deep-sequencing data analysis. Nucleic Acids Res..

[70] Zhang Y (2008). Model-based analysis of ChIP-seq (MACS). Genome Biol..

[71] Stark, R. & Brown, G. DiffBind: differential binding analysis of ChIP–seq peak data. *Bioconductor* http://bioconductor.org/packages/release/bioc/html/DiffBind.html (2012).

[72] Stuart T, Srivastava A, Madad S, Lareau CA, Satija R (2021). Single-cell chromatin state analysis with Signac. Nat. Methods.

[73] Heinz S (2010). Simple combinations of lineage-determining transcription factors prime *cis*-regulatory elements required for macrophage and B cell identities. Mol. Cell.

[74] Vera J (2006). T lymphocytes redirected against the kappa light chain of human immunoglobulin efficiently kill mature B lymphocyte-derived malignant cells. Blood.

[75] Ghosh, J. K., Delampady, M. & Samanta, T. *An Introduction to Bayesian Analysis: Theory and Methods* (Springer, 2006).

[76] Gelman A., et al. *Bayesian Data Analysis* 3rd edition (Chapman & Hall, CRC Texts in Statistical Science, 2013).

[77] Kruschke, J. K. *Doing Bayesian Data Analysis: a Tutorial with R, JAGS and Stan* (Academic Press, 2015).

[78] Schoenfeld DA (1983). Sample-size formula for the proportional-hazards regression model. Biometrics.

