# Supplementary material for: Safety, efficacy and determinants of response of allogeneic CD19-specific CAR-NK cells in CD19+ B cell tumors: a phase 1/2 trial
Source: Nat Med. 2024 Jan 18;30(3):772–84. doi: 10.1038/s41591-023-02785-8 (PMC10957466; doi:10.1038/s41591-023-02785-8)
Supplement: Supplementary file 1 — Supplementary Figs. 1–4 and Supplementary Tables 1 and 2. [file 41591_2023_2785_MOESM1_ESM.pdf]

nature medicine

Article https://doi.org/10.1038/s41591-023-02785-8

Safety, efficacy and determinants of response of allogeneic CD19-specific CAR-NK cells in CD19^{+} B cell tumors: a phase 1/2 trial

In the format provided by the authors and unedited

Safety, efficacy and determinants of response of Allogeneic CD19-specific CAR-NK cells in CD19+ B cell tumors: a phase 1/2 trial

Supplementary Figures and Tables

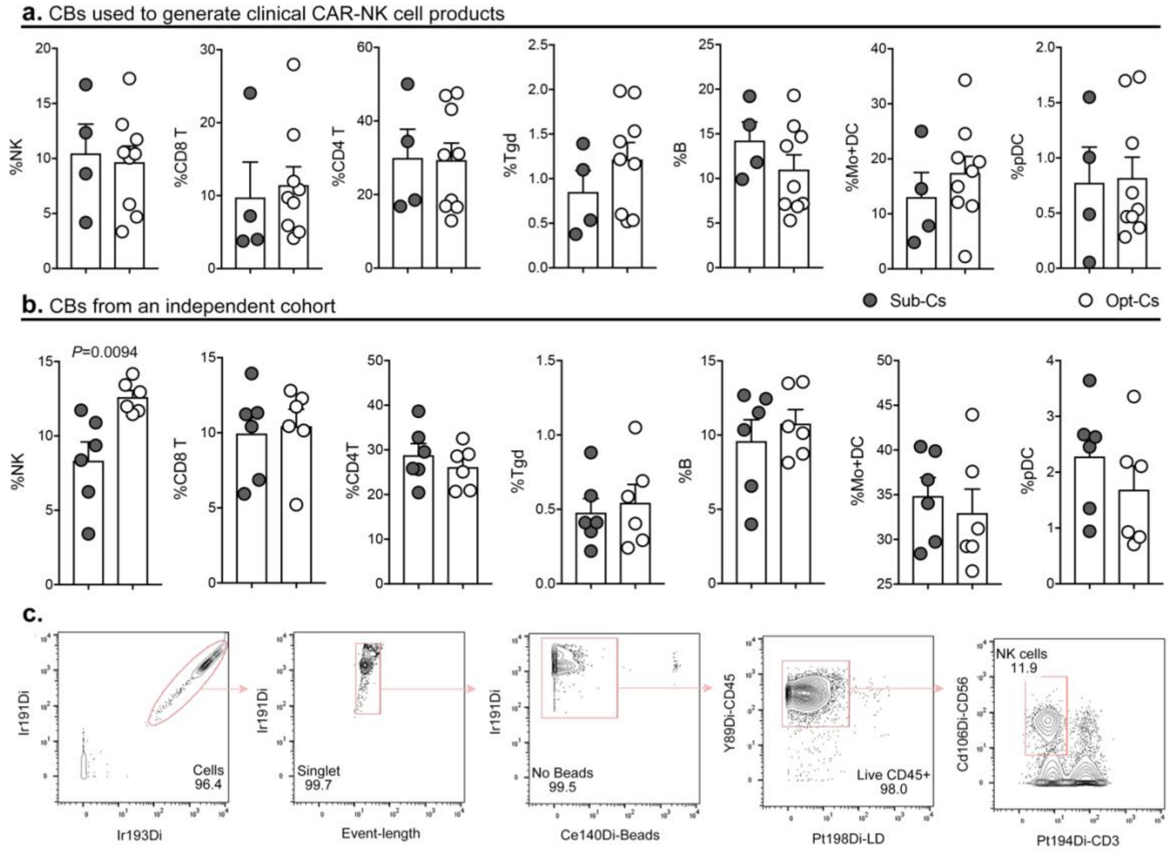

Supplementary Figure 1. Immune composition of cord blood mononuclear cells (CBMCs) used for the generation of the clinical CAR19/IL-15 NK cell products and validated in an independent CBMC cohort. We used cryopreserved CBMCs that were stored in our cord bank from each of the cords used to manufacture the clinical CAR19/IL-15 CB-NK cell product to characterize differences in the composition of the immune subsets between Opt-Cs (n=9 donors) and Sub-Cs (n=4 donors) in the experiment described in panel **a**. For the experiment described in panel **b** we analyzed CBMCs from an independent cohort of CB units from our bank, Sub-Cs (n=6 donors) and Opt-Cs (n=6 donors). (**a** and **b**) Bar plots showing the frequencies of NK cells (CD45+CD56+CD3-), CD8 T cells (CD45+CD56-CD3+CD8+), CD4 T cells (CD45+CD56-CD3+CD4+), gamma-delta T (Tgd) cells (CD45+CD56-CD3+TCRgd+), B cells (CD45+CD56-CD3-CD19+CD20+), monocytes (Mo) + dendritic cells (DC) (CD45+CD56-CD3-CD14+CD11c+), and plasmacytoid DCs (pDC) (CD45+CD56-CD3-CD11c+CD123+). (**c**) Gating strategy for the immunophenotyping of NK cells from CBMCs by CyTOF. Single live cells were identified based

on natural-abundance Iridium selection, bead depletion and Live/Dead separation. Hematopoietic cells within the live population were then selected by gating on hCD45+. Differential expressions of CD56 and CD3 were used to identify NK cells (CD56+CD3-). P-values were determined by two-tailed Student's t test and shown as mean + s.e.m. Non-significant P-values were not added to the figures. Each symbol represents an individual CB unit sample.

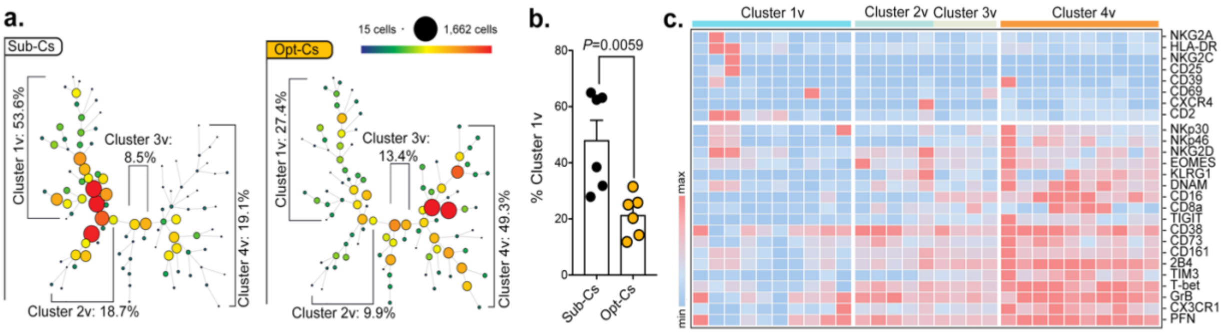

Supplementary Figure 2. Unmanipulated NK cells derived from CBMCs of Opt-Cs or Sub-Cs display distinct phenotypes. To validate the results presented in **Fig. 3**, we used CBMCs from an independent cohort of CBs from our bank. (**a**) SPADE analysis of CyTOF data showing the phenotype of unmanipulated live hCD45$^+$CD56$^+$CD3$^-$ NK cells in CBMCs of Sub-Cs (n=6 donors) or Opt-Cs (n=6 donors). The phenotypic signatures of collected NK cells were evaluated by CyTOF, downsampled to 10,000 cells per sample, pooled and separated into two categories: Sub-Cs vs. Opt-Cs. Clustering by SPADE revealed four main clusters (Clusters 1v-4v). Frequencies of each cluster are indicated; size and color of nodes represent numbers of clustered cells. (**b**) Bar graph shows the percentage (%) of NK cells within Cluster 1v in CBMCs from Sub-Cs vs. Opt-Cs. (**c**) Heatmap representing the expression levels of NK cell markers within the main sub-clusters of Cluster 1v-4v. Each column represents a major node within the SPADE tree clusters. The major nodes are those that are representative of the majority of cells across all conditions. The expression level for each marker is represented on a color scale ranging from the color blue (low) to the color red (high). P-values were determined by two-tailed Student's t test and data shown as mean + s.e.m. Each symbol represents an individual sample.

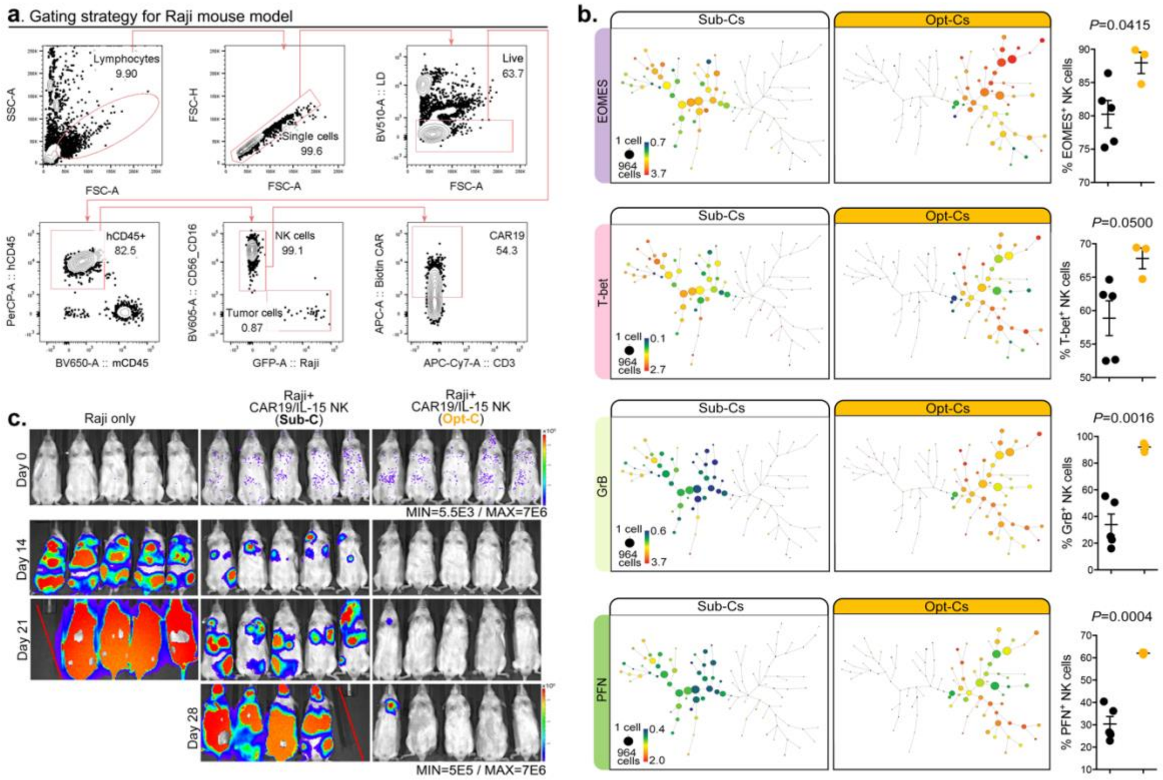

Supplementary Figure 3. CAR-NK cells derived from Opt-Cs show superior anti-tumor effector function in a Raji mouse model. (**a**) Gating strategies for NK cells and tumor cells for the *in vivo* Raji mouse model experiments presented in **Extended Data Fig. 9**. (**b**) SPADE analysis of live hCD45$^+$CD56$^+$CD3$^-$ NK cells in the bone marrow (BM) of mice collected 14 days after CAR19/IL-15 NK-cell injection. The phenotypic signatures of all gated NK cells were evaluated by CyTOF, downsampled to 10,000 cells per sample, pooled and divided into two categories: Sub-Cs vs. Opt-Cs. SPADE analysis was used to visualize the phenotypic differences between the two groups. The mean expression levels for the key NK cell markers EOMES, T-bet, GrB, and PFN are shown for each sub-cluster. The dot plots show the frequencies of NK cells expressing the marker of interest (Sub-Cs; n=5 mice vs. Opt-Cs; n=3 mice). (**c**) Bioluminescence imaging (BLI) corresponding to the Raji mouse model treated with CAR19/IL-15 NK cells described in **Extended Data Fig. 9f**. P-values were determined by two-tailed Student's t test. Data were analyzed by CyTOF and shown as mean ± s.e.m. Each symbol represents an individual sample.

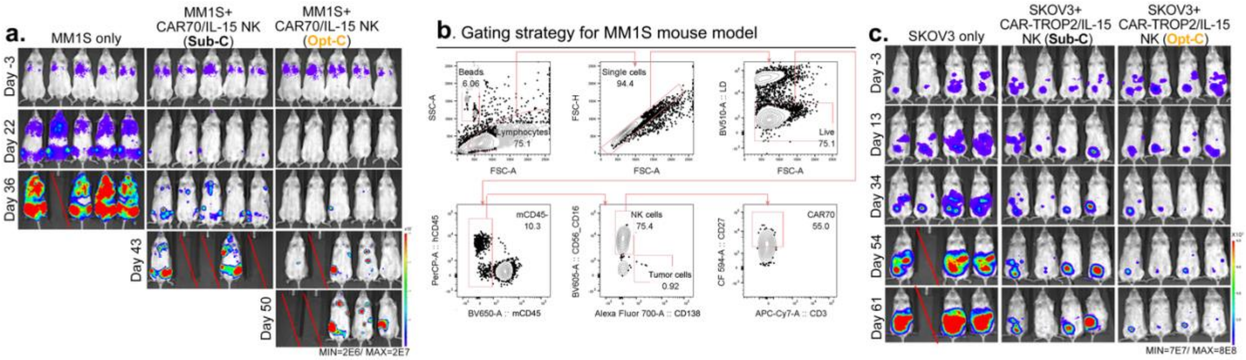

Supplementary Figure 4. CAR-NK cells derived from Opt-Cs show superior anti-tumor effector function in MM1S and SKOV3 mouse models. (**a**) BLI corresponding to the MM1S mouse model treated with CAR70/IL-15 NK cells described in **Extended Data Fig. 9i** (**b**) Gating strategies for NK cells and tumor cells for the *in vivo* MM1S mouse model experiments presented in **Extended Data Fig. 9**. (**c**) BLI corresponding to the SKOV3 mouse model treated with CAR-TROP2/IL-15 NK cells described in **Extended Data Fig. 9n**.

Supplementary Tables

Supplementary Table 1. Patient, disease, donor cord blood unit (CBU) and CAR-NK characteristics in the dose escalation and dose expansion cohorts

**Variable**	**n (%)**	
**Patient characteristics**	**Dose escalation cohort (n=11)**	**Dose expansion cohort (n=26)**
**Age**		
Median (range)	60.0 (49-70)	65.5 (26-79)
< 65 years	9 (81.8)	10 (38.5)
≥ 65 years	2 (18.2)	16 (61.5)
**Sex**		
Male	7 (63.6)	17 (65.4)
Female	4 (36.4)	9 (34.6)
**Race**		
Caucasian	8 (72.7)	23 (88.5)
Hispanic	0 (0.0)	0 (0.0)
Black	0 (0.0)	1 (3.8)
Asian	1 (9.1)	1 (3.8)
Other	2 (18.2)	1 (3.8)
**Weight**		
Median (kg/range)	81.0 (60-138)	77.9 (47-140)
**Diagnosis**		
Indolent lymphoma[1]	1 (9.1)	5 (19.2)
CLL	4 (36.4)	2 (7.7)
CLL-RT	1 (9.1)	4 (15.4)
DLBCL	5 (45.5)	12 (46.2)
Mantle cell lymphoma	0 (0.0)	1 (3.8)
Acute lymphoblastic leukemia	0 (0.0)	1 (3.8)
Lymphoplasmacytic lymphoma	0 (0.0)	1 (3.8)
**Karnofsky (%)**		
Median (range)	100 (90-100)	90 (80-100)
**LDH prior to conditioning chemotherapy**		
Median (range)	422 (188-1715)	241 (141-1765)
LDH>ULN	8 (72.7)	16 (61.5)
**Number of prior therapies**		
Median (range)	4 (3-10)	4 (2-9)
≥3 prior lines of therapy	11 (100.0)	20 (76.9)
**Prior stem cell transplant**		
Autologous	4 (36.4)	5 (19.2)
Allogeneic	0 (0.0)	1 (3.8)
**Disease status**		
Relapsed	7 (63.6)	4 (15.4)
Refractory to most recent therapy	2 (18.2)	14 (53.8)
History of primary refractory disease	2 (18.2)	8 (30.8)
**Disease stage[2]**		
I or II	2 (33.3)	6 (37.5)
III or IV	4 (66.6)	10 (62.5)

Variable	n (%)	
**CAR-NK characteristics**	**Dose escalation cohort (n=11)**	**Dose expansion cohort (n=26)**
**Dose level**		
$1x10^5$ cells/kg	3 (27.3)	0
$1x10^6$ cells/kg	4 (36.4)	0
$1x10^7$ cells/kg	4 (36.4)	11 (42.3)
$8x10^8$ cells flat dose	0	15 (57.7)
**Mean fluorescence intensity**		
Median (range)	10875 (7510-36328)	13278 (4836-31618)
25% percentile	8203	10798
**Transduction efficiency (%)**		
Median (range)	49.0 (22.7-66.45)	77.4 (41.6-91.1)
25% percentile	38.6	65.9
**CD3+ T-cells infused/kg**		
Median (range)	500 (30-8000)	3000 (500-16000)

Variable	n (%)	
**CBU characteristics**	**Dose escalation cohort (n=11)**	**Dose expansion cohort (n=26)**
**HLA allelic match**		
0/6	0	5 (19.2)
1/6	1 (9.1)	8 (30.8)
2/6	1 (9.1)	1 (3.8)
3/6	0	2 (7.7)
4/6	9 (81.8)	10 (38.5)
**KIR ligand mismatch**		
No	8 (72.7)	13 (50)
Yes	3 (27.3)	13 (50)
**Sex**		
Male	6 (54.5)	16 (61.5)
Female	5 (45.5)	10 (38.5)
**CBU race**		
Asian	1 (9.1)	0
Black	1 (9.1)	0
Hispanic	1 (9.1)	10 (38.5)
Caucasian	7 (63.6)	16 (61.5)
Multiple	1 (9.1)	0

**Time from collection to freezing (hours)**<br>Median (range)	24.3 (16.5-42.4)	24.5 (13.4-34.9)
**Pre-freezing CBU viability (%)**<br>Median (range)	99.0 (89-99)	99.0 (91-100)
**Days in culture**<br>Median (range)	15 (15-22)	22 (15-22)
**TNC content (x10^7 cells)**<br>Median (range)<br>25% percentile	124.8 (87.3-248.8)<br>111.9	184.5 (124.1-241.3)<br>162.5
**NRBC content (x10^7 cells)**<br>Median (range)<br>75% percentile	3.2 (1.0-23.8)<br>2.8	5.2 (0.0-19.2)<br>2.7

Abbreviations: CLL: chronic lymphocytic leukemia; CLL-RT: chronic lymphocytic leukemia with Richter's transformation; DLBCL: diffuse large B cell lymphoma; LDH: lactate dehydrogenase; ULN: upper limit of normal; CBU: cord blood unit; TNC: total nucleated cell; NRBC: nucleated red blood cell.

[1]Four patients had follicular lymphoma and two patients had marginal zone lymphoma.

[2]Only for non-Hodgkin's lymphoma (NHL) patients.

Supplementary Table 2. List of samples used in bulk RNA-seq and ATAC-seq analyses

	Sample	Donor	Replicate ID	Cord quality
Bulk RNA-seq	C2-10	EX47-14	3	optimal
	C2-1	EX47-7	1	optimal
	C2-11	EX47-13	1	optimal
	C2-12	EX47-13	2	optimal
	C2-13	EX47-13	3	optimal
	C2-2	EX47-7	2	optimal
	C2-3	EX47-7	3	optimal
	C2-4	EX47-11	1	optimal
	C2-5	EX47-11	2	optimal
	C2-6	EX47-11	3	optimal
	C2-8	EX47-14	1	optimal
	C2-9	EX47-14	2	optimal
	D2-10	EX47-15	2	optimal
	D2-11	EX47-15	3	optimal
	D2-3	EX47-16	1	optimal
	D2-4	EX47-16	2	optimal
	D2-5	EX47-16	3	suboptimal
	D2-6	EX47-17	1	suboptimal
	D2-7	EX47-17	2	suboptimal
	D2-8	EX47-17	3	suboptimal
	D2-9	EX47-15	1	suboptimal
	I1	EX29-1	1	suboptimal
	I2	EX29-1	2	suboptimal
	I3	EX29-2	1	suboptimal
	I4	EX29-2	2	suboptimal
	I5	EX29-2	3	suboptimal
	I7	EX29-4	1	suboptimal
	B11	EX28-1	1	suboptimal
	B21	EX28-1	2	suboptimal
	B31	EX28-3	1	suboptimal
	S4	EX27-4	1	optimal
	S5	EX27-4	2	optimal
Total	32	16		

Bulk ATAC-seq	Sample_O4	EX27-2	1	optimal
	Sample_O5	EX27-2	2	optimal
	Sample_Ple_O7	EX27-4	1	optimal
	Sample_Ple_O8	EX27-4	2	optimal
	Sample_Ple_O9	EX27-4	3	optimal
	Sample_Ple_X1	EX27-4	4	optimal
	Sample_Ple_X2	EX28-1	1	optimal
	Sample_X7	EX29-1	1	optimal
	Sample_O1	EX27-1	1	suboptimal
	Sample_O2	EX27-1	2	suboptimal
	Sample_O3	EX27-1	3	suboptimal
	Sample_O6	EX27-3	1	suboptimal
	Sample_X3	EX28-3	1	suboptimal
	Sample_X4	EX28-3	2	suboptimal
	Sample_X5	EX28-4	1	suboptimal
	Sample_X6	EX28-4	2	suboptimal
	Sample_X8	EX29-4	1	suboptimal
Total	17	7		
