# Supplementary material for: Safety, efficacy and determinants of response of allogeneic CD19-specific CAR-NK cells in CD19+ B cell tumors: a phase 1/2 trial
Source: Nat Med. 2024 Jan 18;30(3):772–84. doi: 10.1038/s41591-023-02785-8 (PMC10957466; doi:10.1038/s41591-023-02785-8)
Supplement: Supplementary file 2 — Reporting Summary [file 41591_2023_2785_MOESM2_ESM.pdf]

nature portfolio

Corresponding author(s): Katayoun Rezvani

Last updated by author(s): Dec 17, 2023

Reporting Summary

Nature Portfolio wishes to improve the reproducibility of the work that we publish. This form provides structure for consistency and transparency in reporting. For further information on Nature Portfolio policies, see our Editorial Policies and the Editorial Policy Checklist.

Statistics

For all statistical analyses, confirm that the following items are present in the figure legend, table legend, main text, or Methods section.

n/a	Confirmed	
☐	☒	The exact sample size (*n*) for each experimental group/condition, given as a discrete number and unit of measurement
☐	☒	A statement on whether measurements were taken from distinct samples or whether the same sample was measured repeatedly
☐	☒	The statistical test(s) used AND whether they are one- or two-sided<br>*Only common tests should be described solely by name; describe more complex techniques in the Methods section.*
☐	☒	A description of all covariates tested
☐	☒	A description of any assumptions or corrections, such as tests of normality and adjustment for multiple comparisons
☐	☒	A full description of the statistical parameters including central tendency (e.g. means) or other basic estimates (e.g. regression coefficient) AND variation (e.g. standard deviation) or associated estimates of uncertainty (e.g. confidence intervals)
☐	☒	For null hypothesis testing, the test statistic (e.g. *F*, *t*, *r*) with confidence intervals, effect sizes, degrees of freedom and *P* value noted<br>*Give P values as exact values whenever suitable.*
☐	☒	For Bayesian analysis, information on the choice of priors and Markov chain Monte Carlo settings
☒	☐	For hierarchical and complex designs, identification of the appropriate level for tests and full reporting of outcomes
☒	☐	Estimates of effect sizes (e.g. Cohen's *d*, Pearson's *r*), indicating how they were calculated

Our web collection on statistics for biologists contains articles on many of the points above.

Software and code

Policy information about availability of computer code

Data collection	The human gene database GeneCard were used for design of CAR constructs. Redcap was used to collect patient data. LSRFortessa™ and X-20 were used to collect flow cytometry data. Helios instrument was used to collect Cytof data. Metabolism assays were performed on Seahorse XFe96 Analyzer. Isoplexis assays were performed using the IsoLight device to scan the IsoCode chips. Luminex assays were performed on the Luminex 200 System. Bulk RNA sequencing was done using Illumina's NextSeq2000 Sequencing System. Bulk ATAC sequencing was done on Illumina NovaSeq6000. IVIS Imaging System were used for bioluminescence imaging of mice.
Data analysis	FlowJo version 10.8.1, living image V4.4, Microsoft Excel for Mac 2011, GraphPad Prism version 7, Cytobank v10.3, Morpheus (online tool no version available), Helios 6.5.358 acquisition software, IsoSpeak software v2.8.0.0, IncuCyte Live-Cell Analysis System 2022B Rev2, Agilent Seahorse XF Pro Analyzer, Bio-Plex Version 6.2, SPSS Version 26.0, R version 4.2.1, JAGS version 4.3.1. For bulk RNAseq, sequence read data were processed and converted to FASTQ format for downstream analysis by Illumina BaseSpace software, BCL Convert 3.8.4. Differential expression analysis was performed using DESeq2 v1.30.1. Signac and Seurat were used for downstream analysis of ATACseq. No custom code was developed in this study.

For manuscripts utilizing custom algorithms or software that are central to the research but not yet described in published literature, software must be made available to editors and reviewers. We strongly encourage code deposition in a community repository (e.g. GitHub). See the Nature Portfolio guidelines for submitting code & software for further information.

Data

Policy information about availability of data

All manuscripts must include a data availability statement. This statement should provide the following information, where applicable:

- Accession codes, unique identifiers, or web links for publicly available datasets
- A description of any restrictions on data availability
- For clinical datasets or third party data, please ensure that the statement adheres to our policy

ATAC and RNA-seq data are available through the Gene Expression Omnibus (https://www.ncbi.nlm.nih.gov/geo/) under accession number GSE233149.
The pySCENIC database is available through the following link of the pySCENIC cistarget database: https://resources.aertslab.org/cistarget/databases/homo_sapiens/hg38/refseq_r80/mc9nr/gene_based/.
The data reported in this article are commercially sensitive and not publicly available. To the extent allowed, the authors will provide access to deidentified participant-level data underlying the data presented in this article to researchers who provide a methodologically sound proposal for academic purposes to interpret, verify and extend research in the article that does not violate privacy, data encumbrance, intellectual property or other legal, regulatory, or contractual confidentiality obligations, beginning 12 months after article publication. Data provided will be subject to a data use agreement. Researchers should contact the corresponding author when applying for data access. Response to external data requests will be within a reasonable timeframe of a few weeks to months depending on the nature of the request. Use of data will be restricted to the agreed purpose.
Source data are provided with this manuscript.

Research involving human participants, their data, or biological material

Policy information about studies with human participants or human data. See also policy information about sex, gender (identity/presentation), and sexual orientation and race, ethnicity and racism.

Reporting on sex and gender	Research findings do not apply to one sex or gender.
Reporting on race, ethnicity, or other socially relevant groupings	Research findings do not apply to any race, ethnicity or socially relevant group.
Population characteristics	Detailed population demographics, disease and treatment characteristics are included in Table 1 and Supplementary Table 1 of the manuscript.
Recruitment	Patients with relapsed refractory CD19+ B-cell malignancies were referred by various departments at The University of Texas MD Anderson Cancer Center for screening for eligibility to be enrolled on the study. If eligible as detailed below, patients were enrolled without bias regarding gender, ethnicity, disease characteristics, or other parameters.<br>Eligibility criteria: Patients 7-80 years of age with relapsed/refractory CD19-positive B-cell malignancies, a Karnofsky performance status of >70% and an adequate organ function were eligible. Patients must have been at least three weeks from the last cytotoxic chemotherapy or at least three days from tyrosine kinase inhibitors or other targeted therapies.<br>Exclusion criteria included: 1) pregnancy, 2) positive serology for HIV, 3) uncontrolled infections, 4) grade III or higher toxicities from prior therapies, 5) active neurological disorders, and 6) receipt of concomitant investigational therapies. Prior CD19 targeting therapy was an exclusion criteria for the second phase of the study.
Ethics oversight	The study was approved by the institutional review board at The University of Texas MD Anderson Cancer Center and conducted according to the declaration of Helsinki. Written informed consent was obtained from each patient. The study was overseen by the External Data Safety Monitoring Board of MD Anderson Cancer Center.

Note that full information on the approval of the study protocol must also be provided in the manuscript.

Field-specific reporting

Please select the one below that is the best fit for your research. If you are not sure, read the appropriate sections before making your selection.

☒ Life sciences ☐ Behavioural & social sciences ☐ Ecological, evolutionary & environmental sciences

For a reference copy of the document with all sections, see nature.com/documents/nr-reporting-summary-flat.pdf

Life sciences study design

All studies must disclose on these points even when the disclosure is negative.

Sample size	For the preclinical experiments, sample sizes were estimated based on preliminary experiments. We made the effort to achieve a minimum sample size of n=4 to 10 mice per treatment group which proved to be sufficient to reproducibly observe statistically significant differences. Power calculations predicted at least 80% power to detect a relative hazard ratio of 4.3-6 between two groups at the significance level of 0.05. The clinical trial was a phase I/II study where patient enrollment was determined using an eff-tox Bayesian model as described in the method section. The statistical rationale for the sample size of patients enrolled on the trial was not based on a power computation. Rather, the reliability of Bayesian posterior estimators of Probability (efficacy) and Probability (toxicity) were quantified by assuming a noninformative prior for each probability and computing a posterior 95% credible interval (CrI).

Data exclusions

No data were excluded.

Replication

All in vitro and in vivo experiments were repeated from different cord blood donors in independent experiments. In vivo experiments were performed independently. Unless otherwise specified, experiments were replicated at least 2-3 times. All attempts at replication were successful. Efficacy of CAR-NK cell treatment varied between suboptimal and optimal cords.

Randomization

For in vitro data, primary cells were obtained from anonymous healthy donors (MD Anderson CB bank). For in vivo data, all mice were imaged routinely pre and post CAR-NK cell infusion. All in vivo experiments were performed using 9-10 week old NOD.Cg-PrkdcscidIl2rgtmWjl/SzJ (NSG) mice purchased from Jackson Laboratory. All comparisons were between identical batches. No randomization methods were required. We have included appropriate controls including positive and negative controls. We ensured equal tumor burden in each group of mice at baseline before treatment.
The clinical study was a Phase I/II non-randomized clinical study. As a result no randomization was performed.

Blinding

For in vivo experiments, mice were injected and imaged by an operator who was blinded to treatment groups and data were analyzed by an investigator who was blinded to the treatment groups. For in vitro functional studies and incucyte based cytotoxicity assays comparing NK cells from optimal vs. suboptimal cords, analysis was done blindly by 2 independent investigators, with the investigators blinded to the cord quality. No blinding methods were used for other experiments (transciptomics and epigenetics) as analyses were done by Bioinformatic experts who had access to the full sample labeling to avoid any mix ups or confusions. Blinding was not applied to the clinical study as this consisted of a phase I non-randomized study.

Reporting for specific materials, systems and methods

We require information from authors about some types of materials, experimental systems and methods used in many studies. Here, indicate whether each material, system or method listed is relevant to your study. If you are not sure if a list item applies to your research, read the appropriate section before selecting a response.

Materials & experimental systems

n/a	Involved in the study
☐	☒ Antibodies
☐	☒ Eukaryotic cell lines
☒	☐ Palaeontology and archaeology
☐	☒ Animals and other organisms
☐	☒ Clinical data
☒	☐ Dual use research of concern
☒	☐ Plants

Methods

n/a	Involved in the study
☒	☐ ChIP-seq
☐	☒ Flow cytometry
☒	☐ MRI-based neuroimaging

Antibodies

Antibodies used

For flow cytometry, antibodies used were the following: Live Dead-BV510 (Invitrogen, 1:200, cat# L34966A), Human CD45-PerCP (Biolegend, HI30, 1:50, cat# 304026), Mouse CD45-BV650 (Biolegend, 30-F11, 1:50, cat# 103151), Human CD56-BV605 (Biolegend, 5.1H11, 1:50, cat# 362538), Human CD16-BV605 (Biolegend, 3G8, 1:50, cat# 302040), Human CD3-APCY7 (Biolegend, HIT3a, 1:100, cat# 300318), Human CD19-PECY7 (BD Biosciences, SJ25C1, 1:50, cat# 557835), Human CD20-AF700 (BD Biosciences, 2H7, 1:50, cat# 560631), Anti Biotin-PE (Miltenyi Biotec, Bio3-18E7, 1:20, cat# 130-113-291), Anti Biotin-APC (Miltenyi Biotec, REA746, 1:50, cat# 130-110-952), CD19 CAR Detection reagent-Unconjugated (Miltenyi Biotec, 1:50, cat# 130-129-550), goat anti-human IgG-AF647 (H +L; Jackson ImmunoResearch, cat# 09-605-088); Human CD27-PECF594 (BD Biosciences, M-T271, 1:50, cat# 562297), Human CD70-PECY7 (Biolegend, 113-16, 1:50, cat# 355112), Human BCMA-PE (Miltenyi Biotec, REA315, 1:50, cat# 130-110-970), Human CD138-AF700 (BD Biosciences, MI15, 1:50, cat# 566050), TROP2-PE (Biolegend, NY18, 1:50, cat# 363804), Anti-His-APC (Biolegend, J095G46, 1:50, cat# 362605).

For Cytof, antibodies used included the following:
CD45 (Standard Biotools, HI30, 89Y, 1:200, cat# 3089003B), CCR6 (Miltenyi Biotec, REA190, 141Pr, 1:125, cat# 130-108-023), EOMES (Invitrogen, WD1928, 142Nd, 1:200, cat# 14-4877-82), KIR2DL4 (Miltenyi Biotec, REA768, 143Nd, 1:250, cat# 130-126-474), KIR3DL1 (BD Pharmingen, DX9, 144Nd, 1:300, cat# 555964), CD70 (Biolegend, 113-16, 145Nd, 1:500, cat# 355102), KIR2DL5 (Miltenyi Biotec, REA955, 146Nd, 1:125, cat# 130-126-477), NKG2C (Miltenyi Biotec, REA205, 147Sm, 1:125, cat# 130-122-278), TRAIL (Miltenyi Biotec, REA1113, 148Nd, 1:125, cat# 130-126-490), CD25 (Standard Biotools, 2A3, 149Sm, 1:125, cat# 3149010B), CD69 (Miltenyi Biotec, REA824, 150Nd, 1:5000, cat# 130-124-326), 2B4 (Miltenyi Biotec, REA112, 151Eu, 1:5000, cat# 130-124-523), Granzyme B (GrB; Miltenyi Biotec, REA226, 152Sm, 1:5000, cat# 130-108-055), TIM3 (Miltenyi Biotec, REA635, 153Eu, 1:125, cat# 130-122-333), CX3CR1 (Miltenyi Biotec, REA385, 154Sm, 1:125, cat# 130-122-286), KIR2DL3 (Miltenyi Biotec, REA147, 155Gd, 1:200, cat# 130-122-280), CXCR3 (Standard Biotools, G025H7, 156Gd, 1:200, cat# 3156004B), OX40 (Miltenyi Biotec, REA621, 158Gd, 1:125, cat# 130-095-212), Perforin (PFN; Miltenyi Biotec, REA1061, 159Tb, 1:5000, cat# 130-126-480), T-bet (Standard Biotools, 4B10, 160Gd, 1:250, cat# 3160010B), TIGIT (Miltenyi Biotec, REA1004, 161Dy, 1:125, cat# 130-122-310), Ki67 (Standard Biotools, B56, 162Dy, 1:250, cat# 3162012B), KIR2DL1 (Miltenyi Biotec, REA284, 163Dy, 1:250, cat# 130-122-279), KIR2DS1 (R&D Systems, 1127B, 164Dy, 1:250, cat# MAB8887), PD1 (Miltenyi Biotec, PD1.3.1.3, 165Ho, 1:125, cat# 130-096-168), NKG2D (Miltenyi Biotec, REA797, 166Er, 1:300, cat# 130-122-332), CD38 (Miltenyi Biotec, REA572, 167Er, 1:500, cat# 130-122-307), CD73 (Standard Biotools, AD2, 168Er, 1:100, cat# 3168015B), CD39 (Miltenyi Biotec, MZ18-23C8, 169Tm, 1:200, cat# 130-093-506), CD161 (Miltenyi Biotec, REA631, 170Er, 1:500, cat# 130-122-347), DNAM (Miltenyi Biotec, REA1040, 171Yb, 1:250, cat# 130-126-485), KLRG1 (Miltenyi Biotec, REA261, 172Yb, 1:125, cat# 130-126-458), CXCR4 (Standard Biotools, 12G5, 173Yb, 1:200, cat# 3173001B), KIR2DS4 (Miltenyi Biotec, REA860, 174Yb, 1:250, cat# 130-122-328), LAG3 (Miltenyi Biotec, REA351, 175Lu, 1:125, cat# 130-124-529), ICOS (Miltenyi Biotec,

REA192, 176Yb, 1:200, cat# 130-122-304), CD16 (Standard Biotools, 3G8, 209Bi, 1:200, cat# 3209002B), CD57 (Miltenyi Biotec, REA769, 115In, 1:500, cat# 130-124-525), CD3 (Miltenyi Biotec, REA613, 194Pt, 1:250, cat# 130-122-282), NKG2A (Miltenyi Biotec, REA110, 195Pt, 1:500, cat# 130-122-329), HLA-DR (Miltenyi Biotec, REA805, 196Pt, 1:250, cat# 130-122-299), LD (Standard Biotools, Cisplatin, 198Pt, 2.5 µM), CD56 (Miltenyi Biotec, REA196, 106Cd, 1:200, cat# 130-108-016), CAR (Miltenyi Biotec, REA1298, 110Cd, 1:50, cat# 130-127-984), CD2 (Miltenyi Biotec, REA972, 111Cd, 1:300, cat# 130-122-348), CD8 (Miltenyi Biotec, REA734, 112Cd, 1:250, cat# 130-122-281), NKP30 (Miltenyi Biotec, AF29-4D12, 113Cd, 1:200, cat# 130-092-554), NKP46 (Miltenyi Biotec, REA808, 114Cd, 1:125, cat# 130-124522), NKP44 (Miltenyi Biotec, REA1163, 116Cd, 1:125, cat# 130-126-465), CD36 (Miltenyi Biotec, REA760, 142Nd, 1:300, cat# 130-124-322), CD127 (Standard Biotools, A019D5, 143Nd, 1:200, cat# 3143012B), CD11b (Standard Biotools, ICRF44, 144Nd, 1:250, cat# 3144001B), CD62L (Miltenyi Biotec, REA615, 145Nd, 1:200, cat# 130-122-326), CD64 (Miltenyi Biotec, REA978, 148Nd, 1:250, cat# 130-124-325), CD86 (Miltenyi Biotec, REA968, 150Nd, 1:125, cat# 130-122-334), CD123 (Miltenyi Biotec, REA918, 151Eu, 1:250, cat# 130-122-297), TCRgd (Miltenyi Biotec, REA591, 152Sm, 1:250, cat# 130-122-291), CD27 (Miltenyi Biotec, REA499, 155Gd, 1:250, cat# 130-122-295), CCR4 (Miltenyi Biotec, REA279, 158Gd, 1:125, cat# 130-122-323), CD11c (Standard Biotools, Bu15, 159Tb, 1:250, cat# 3159001B), CD80 (Standard Biotools, 2D10.4, 161Dy, 1:125, cat# 3161023B), CD66B (Standard Biotools, 80H3, 162Dy, 1:250, cat# 3162023B), TCR Va7.2 (Miltenyi Biotec, REA179, 163Dy, 1:200, cat# 130-126-467), CD45RO (Miltenyi Biotec, REA611, 164Dy, 1:200, cat# 130-124-323), CD163 (Standard Biotools, GHI/61, 165Ho, 1:200, cat# 3165017B), CCR7 (Miltenyi Biotec, REA546, 167Er, 1:200, cat# 130-122-300), CD45RA (Miltenyi Biotec, REA562, 169Tm, 1:200, cat# 130-122-292), CXCR5 (Miltenyi Biotec, REA103, 171Yb, 1:300, cat# 130-122-325), iNKT (Biolegend, 6B11, 173Yb, 1:200, cat# 342902), CD95 (Standard Biotools, DX2, 175Lu, 1:250, cat# 3164008B), CD19 (Miltenyi Biotec, REA675, 110Cd, 1:200, cat# 130-122-301), CD4 (Miltenyi Biotec, REA623, 111Cd, 1:300, cat# 130-122-238), CD15 (BD Pharmingen, HI98, 113Cd, 1:500, cat# 555400), CD14 (Miltenyi Biotec, REA599, 114Cd, 1:200, cat# 130-122-290), CD20 (Miltenyi Biotec, REA780, 116Cd, 1:200, cat# 130-124-537), GFP (Biolegend, FM264G, 144Nd, 1:250, cat# 338002), CD81 (Miltenyi Biotec, REA513, 145Nd, 1:250, cat# 130-124-538), PANKIR (R&D, 180704, 153Eu, 1:300, cat# MAB1848), mCD45 (Biolegend, 30-F11, 154Sm, 1:125, cat# 103102), PFN (Standard Biotools, B-D48, 196Pt, 1:200, cat# 3196002B), GrB (Standard Biotools, GB11, 198Pt, 1:200, cat# 3198002B).

Validation

All used antibodies were titrated and the best titrations were determined based on the signal of the positive controls (either PBMCs or activated immune effector cells) as well as negative controls (such as cells stained with isotype or FMO controls). All the antibodies are validated for use in flow cytometry and CyTOF as shown in the manufacturer's website. Antibodies that were conjugated with metals were reconstituted in buffer with no BSA or other carrier proteins that could interfere with conjugation. All used antibodies are commercially available.

Eukaryotic cell lines

Policy information about cell lines and Sex and Gender in Research

Cell line source(s)

Cell lines of Raji (CCL-86), MM1S (CRL-2974), SKOV3 (HTB-77), K562 (CRL-3344) and 293T (CRL-3216) were obtained from the American Type Culture Collection (ATCC).

Authentication

The American Type Culture Collection (ATCC) uses morphology, karyotyping, PCR and STR assays to authenticate cell lines such as the Raji cell line. Morphology and properties pertinent to the experiments as antigens expression were confirmed routinely by flow cytometry.

Mycoplasma contamination

All cell lines were routinely tested for mycoplasma contamination and found to be negative.

Commonly misidentified lines
(See ICLAC register)

No commonly mis-identified cell lines were used.

Animals and other research organisms

Policy information about studies involving animals; ARRIVE guidelines recommended for reporting animal research, and Sex and Gender in Research

Laboratory animals

NSG mice were 9-10 weeks old for the genotype NOD.Cg-Prkdc<scid>Il2rg<tml Wjl>/SzJ (Jackson laboratory). Mice were maintained under specific-pathogen-free conditions, with a 12-hour night/day cycle of light, and at a stable ambient temperature with 40-70% relative humidity.

Wild animals

This study did not involve wild animals.

Reporting on sex

Sex was not considered in the study design.

Field-collected samples

This study did not involve field-collected samples.

Ethics oversight

All experiments were performed in accordance with American Veterinary Medical Association (AVMA) and NIH recommendations under protocols approved by the MD Anderson Cancer Center Institutional Animal Care and Use Committee (protocol number 00000889-RN02).

Note that full information on the approval of the study protocol must also be provided in the manuscript.

Clinical data

Policy information about clinical studies

All manuscripts should comply with the ICMJE guidelines for publication of clinical research and a completed CONSORT checklist must be included with all submissions.

Clinical trial registration: NCT03056339

Study protocol: The full trial protocol will be available upon request.

Data collection: Patients were treated between June 2017 and June 2021 in a FDA-approved clinical trial. Data were collected prospectively and entered into electronic case report forms by data managers at MD Anderson Cancer Center. The integrity of the data was reviewed at regular intervals by the investigators and data managers. Data were stored using MD Anderson issued devices. The quality and accuracy of the data was audited at regular intervals by the MD Anderson Cancer Center IND office.

Outcomes: Outcomes were:
1. Primary outcomes.
a- Safety. Toxicity is defined as grade 3 or 4 GVHD within 40 days of CAR-NK cell infusion or cytokine release syndrome (CRS) within 2 weeks of CAR-NK cell infusion requiring transfer to intensive care.
b- Efficacy is defined as the patient being alive and in at least partial remission at day 30 post NK cell infusion.
2. Secondary outcomes. Secondary outcomes include progression-free survival (PFS) time, overall survival (OS) time, and response at day 100.
Clinical responses to therapy for CLL and NHL were based on the Lugano and iWCLL 2018 criteria, respectively. Overall response (OR) represents the combination of partial response (PR) and complete response (CR). Day +30 OR was defined as the achievement of PR or CR at any time within 30 days after the infusion. One-year CR was defined as the achievement of CR at any time within 1 year after the infusion. All patients who achieved CR during follow-up were in PR at day 30.

Plants

Seed stocks: *Report on the source of all seed stocks or other plant material used. If applicable, state the seed stock centre and catalogue number. If plant specimens were collected from the field, describe the collection location, date and sampling procedures.*

Novel plant genotypes: *Describe the methods by which all novel plant genotypes were produced. This includes those generated by transgenic approaches, gene editing, chemical/radiation-based mutagenesis and hybridization. For transgenic lines, describe the transformation method, the number of independent lines analyzed and the generation upon which experiments were performed. For gene-edited lines, describe the editor used, the endogenous sequence targeted for editing, the targeting guide RNA sequence (if applicable) and how the editor was applied.*

Authentication: *Describe any authentication procedures for each seed stock used or novel genotype generated. Describe any experiments used to assess the effect of a mutation and, where applicable, how potential secondary effects (e.g. second site T-DNA insertions, mosiacism, off-target gene editing) were examined.*

Flow Cytometry

Plots

Confirm that:

☒ The axis labels state the marker and fluorochrome used (e.g. CD4-FITC).

☒ The axis scales are clearly visible. Include numbers along axes only for bottom left plot of group (a 'group' is an analysis of identical markers).

☒ All plots are contour plots with outliers or pseudocolor plots.

☒ A numerical value for number of cells or percentage (with statistics) is provided.

Methodology

Sample preparation: The clinical CB units for CAR-NK production were obtained from the MD Anderson Cord Blood Bank. CB was collected after informed consent from mothers at several hospitals and shipped to the MD Anderson Cord Bank for processing and cryopreservation following standard operating procedures (SOPs). The time from collection-to-cryopreservation was the time from collection of CB at mother's bedside to the time the cord was cryobanked. The CAR-NK cells were manufactured in the MD Anderson Cancer Center Good Manufacturing Practice (GMP) facility. Briefly, the cord unit was thawed in a water bath, and NK cells were purified by CD3, CD19 and CD14 negative selection (Miltenyi beads) and cultured in the presence of engineered K562 feeder cells expressing membrane-bound IL-21 and 4-1BB ligand plus exogenous IL-2 (200 U/ml). On day 6 of culture, cells were transduced with a retroviral vector encoding the anti-CD19 CAR, IL-15 and iC9 genes, generously provided by Dr. Gianpietro Dotti (University of North Carolina, USA). The cells were expanded for an additional nine days and harvested for fresh infusion on day 15. For a subset of patients (n=17), the products were expanded for a total of 22 days.

Detailed sample preparation are described in the methods section.

Instrument: LSRFortessa™ and X-20 were used to collect flow cytometry data.

Software	Software to analyse flow data: FlowJo (10.8.1)
Cell population abundance	No cell population abundance is predefined and applied to in all in vitro and in vivo samples.
Gating strategy	In the Raji mouse model, the NK cell population was identified by first gating on lymphocytes using forward and side scatters. We next gated on singlets, followed by live cells defined as Live Deadlow . Human NK cells were identified by first gating on hCD45+mCD45- followed by CD16+CD56+GFP-cells. CAR19+ NK cells were identified using conjugated goat anti-human lgG; fluorescence minus one (FMO) or NT-NK cells were used as controls. To identify Raji cells, we first gated on the hCD45 +mCD45- population, followed by CD16-CD56-CD19+GFP+ cells. In the MM1S mouse model, the NK cell population was identified by first gating on lymphocytes using forward and side scatters, then on singlets, followed by Live Deadlow, then mCD45-CD138- and finally CD16+CD56+cells. CAR70+ NK cells were identified as CD16+CD56+CD27+, with FMO or NT-NK cells used as controls. MM1S cells were gated from the Live Deadlow population and identified as hCD45-CD138+.<br><br>Detailed gating strategies are explained in the flow cytometry methods section. Further gating strategies will be provided upon request.

☒ Tick this box to confirm that a figure exemplifying the gating strategy is provided in the Supplementary Information.
